# A Preliminary Investigation of Individual Differences in Subjective Responses to D-Amphetamine, Alcohol, and Delta-9-Tetrahydrocannabinol Using a Within-Subjects Randomized Trial

**DOI:** 10.1371/journal.pone.0140501

**Published:** 2015-10-29

**Authors:** Margaret C. Wardle, Benjamin A. Marcus, Harriet de Wit

**Affiliations:** 1 Center for Neurobehavioral Research on Addiction, Department of Psychiatry and Behavioral Sciences, University of Texas Health Science Center at Houston. Houston, TX, United States of America; 2 Human Behavioral Pharmacology Laboratory, Department of Psychiatry and Behavioral Neuroscience, MC 3077, The University of Chicago, Chicago, IL, United States of America; Erasmus University Rotterdam, NETHERLANDS

## Abstract

Polydrug use is common, and might occur because certain individuals experience positive effects from several different drugs during early stages of use. This study examined individual differences in subjective responses to single oral doses of d-amphetamine, alcohol, and delta-9-tetrahydrocannabinol (THC) in healthy social drinkers. Each of these drugs produces feelings of well-being in at least some individuals, and we hypothesized that subjective responses to these drugs would be positively correlated. We also examined participants’ drug responses in relation to personality traits associated with drug use. In this initial, exploratory study, 24 healthy, light drug users (12 male, 12 female), aged 21–31 years, participated in a fully within-subject, randomized, counterbalanced design with six 5.5-hour sessions in which they received *d*-amphetamine (20mg), alcohol (0.8 g/kg), or THC (7.5 mg), each paired with a placebo session. Participants rated the drugs’ effects on both global measures (e.g. feeling a drug effect at all) and drug-specific measures. In general, participants’ responses to the three drugs were unrelated. Unexpectedly, “wanting more” alcohol was inversely correlated with “wanting more” THC. Additionally, in women, but not in men, “disliking” alcohol was negatively correlated with “disliking” THC. Positive alcohol and amphetamine responses were related, but only in individuals who experienced a stimulant effect of alcohol. Finally, high trait constraint (or lack of impulsivity) was associated with lower reports of liking alcohol. No personality traits predicted responses across multiple drug types. Generally, these findings do not support the idea that certain individuals experience greater positive effects across multiple drug classes, but instead provide some evidence for a “drug of choice” model, in which individuals respond positively to certain classes of drugs that share similar subjective effects, and dislike other types of drugs.

**Trial Registration:**

ClinicalTrials.gov NCT02485158

## Introduction

Most drug users use more than one pharmacological class of drugs, and many develop dependence on several drugs [1]—these individuals are termed polydrug users. Polydrug use is common across many drug classes, including stimulants, sedatives, alcohol and marijuana [2–4].. This suggests that there may be underlying biological mechanisms that promote a risk for drug use regardless of drug class [5]. One mechanism that may put certain people at risk for using multiple drugs is that they are inherently susceptible to the reinforcing effects of drugs, regardless of drug class.

One potential reinforcing effect of drugs is the subjective feelings of euphoria, liking or “high” that the drugs produce. Self-reported liking of subjective drug effects appears to predict use and abuse [6]. For example, studies indicate that early positive subjective reactions to marijuana and alcohol predict greater use later in life [7–10]. In addition, in laboratory studies when volunteers are allowed to choose a drug (e.g., alcohol (ALC), amphetamine (AMP) or diazepam) over a placebo, those who choose the drug typically report more positive subjective experiences from the preferred drug [11–14]. Overall, these studies suggest that positive subjective responses to drugs, such as “liking” the drug, during the early stages of drug use, may form part of a liability for future drug use and abuse.

To the extent that pleasurable subjective responses to drugs of abuse increase the risk for excessive use, increased pleasurable responses to several different classes of drugs may contribute to polydrug use. The idea that some individuals may respond more positively to many different types of drugs can be tested by measuring the subjective and behavioral effects of different drugs in the same group of recreational drug users. Our laboratory and others have conducted several studies related to this question, examining relationships between responses to ALC and other drugs including AMP, diazepam, and triazolam [15–18]. Although these studies provide initial support for the hypothesis that there are commonalities in responses across drug classes in humans, most controlled studies have examined only pairs of drugs, usually pairs with some similarities in their subjective effects (e.g. stimulant responses to ALC and AMP). Two studies examined responses to three different drugs. However, one of these studies (comparing ALC, tobacco, and marijuana) used retrospective reports of drug use experiences [19], and the other (comparing ALC, caffeine, and nicotine) examined the responses to three drugs all with similar stimulant effects [18]. Thus, there has not yet been a controlled laboratory study examining individuals’ subjective responses to more than two classes of drugs with differing subjective effects.

In this initial exploratory study, we examined acute responses of the same individuals to three distinct drugs of abuse: AMP, ALC, and delta-9-tetrahydrocannabinol (THC, a primary psychoactive constituent of marijuana). These drugs act initially on different neurotransmitter systems, via dopaminergic, GABAergic and other, and endocannabinoid pathways, respectively [20–22], and they elicit different subjective effects. However, they also all have the capacity to release dopamine in the mesolimbic pathway, a hypothesized common neural substrate for the reinforcing effects of all drugs of abuse [23]. By choosing this set of drugs, we thus allowed for both unique and shared drug responses. Further, these three drugs are commonly involved in polydrug use [2–4]. For example, college students who abuse stimulant drugs are six times more likely to report frequent heavy drinking than those who do not abuse stimulants [24]. Also, simultaneous use of alcohol and marijuana is especially prevalent [4, 25, 26]. In our study, healthy social drinkers received single doses of AMP, ALC, and THC in a placebo-controlled, double-blind study. We predicted that individuals would exhibit positively correlated subjective responses to the three drugs on non-drug-specific ratings of liking, disliking, and feeling high. Such commonalities in subjective experiences across drug types would suggest that some individuals are especially sensitive to the positive subjective effects of drugs, regardless of drug class, which might in turn represent a susceptibility to the development of polydrug use and dependence.

Secondarily, we examined whether certain personality traits influence subjective drug responses across drug classes. We examined three broad personality traits previously implicated in responses to individual drugs in this study: social potency, which predicts AMP-associated euphoria [27], impulsivity, which has predicted enhanced responses to AMP, ALC, and THC in separate samples [27–29], and negative emotionality, which is associated with problematic drinking and earlier initiation of marijuana abuse [30–32]. To explore the potential influence of personality traits on subjective effects across drug classes, we examined the relationship between these three personality traits and subjective responses to AMP, ALC, and THC in the same individuals.

## Methods

### 2.1 Study Design

In a within-subjects design, healthy adult participants attended six 5.5-hour sessions in which they received 20 mg AMP, 0.8 g/kg ALC, and 7.5 mg THC, alternating with three placebo sessions. Drugs were administered under double blind and double dummy conditions (i.e., both a drink and capsule were administered at every session). The order of the AMP, ALC, and THC sessions and whether the alternating placebo/drug sequence began with a drug or placebo session were counterbalanced, resulting in 12 possible conditions. Two random sequences of the conditions (one for male and one for female participants) were generated by the first author using www.random.org, and participants were assigned in order of recruitment to these conditions until all conditions were filled. To blind research assistants to the sequence, condition assignments were made by the first author, who had no participant contact. The same author also made up all capsules and drinks for the participants (see 2.3) to maintain the blind for the research assistants, who ran all sessions and conducted all data management prior to final analyses. Participants’ subjective and behavioral responses to the drugs were assessed at regular intervals during each session, as detailed in Procedure (see 2.4).

### 2.2 Participants

**Fig 1:**
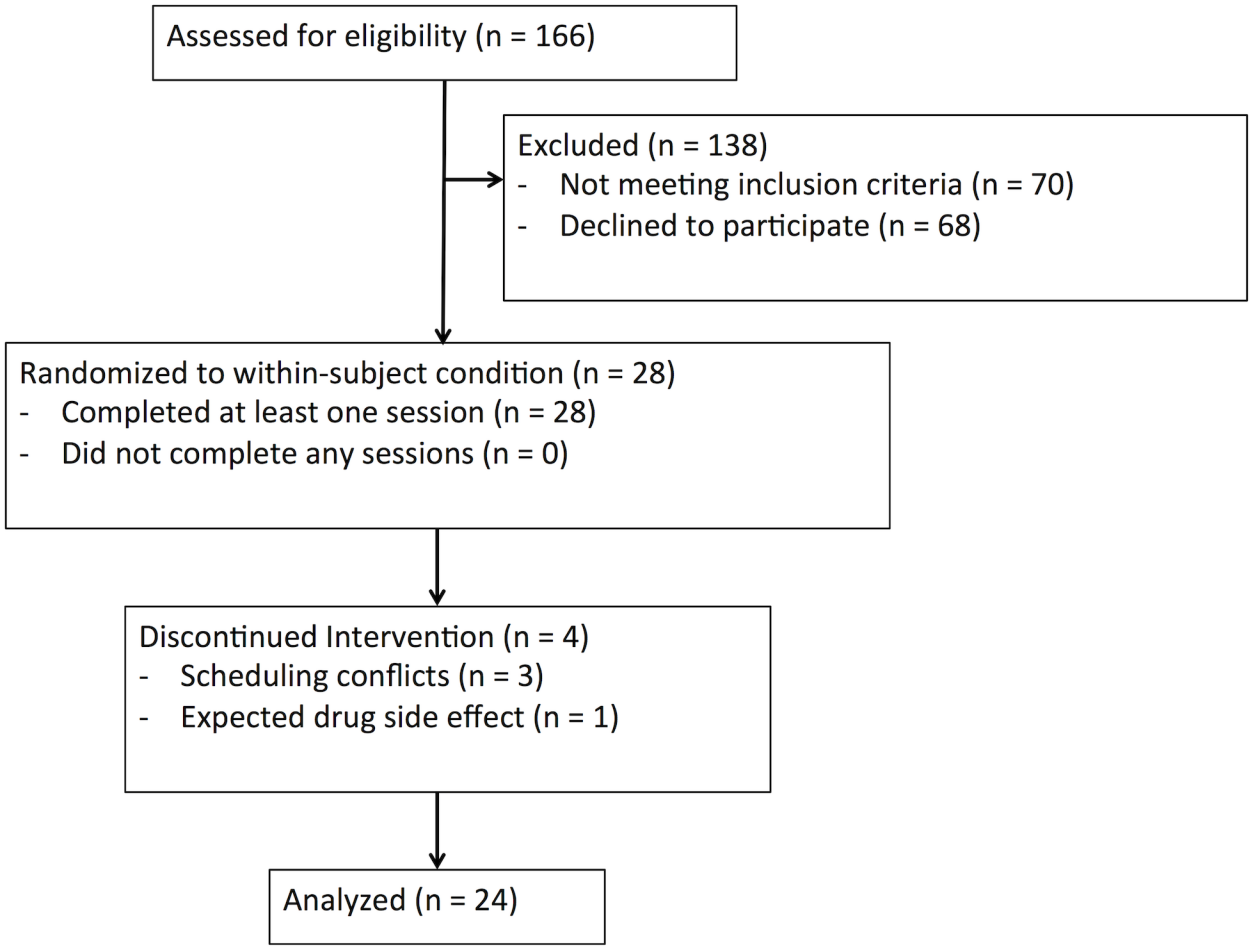
CONSORT Diagram. Number of participants screened, randomized and completing the study, with reasons for exclusion and discontinuation.

Healthy social drinkers (n = 24, 12 male), ages 21–31, were recruited from the Chicago area through flyers and online advertisements, from June 2013 through February 2014. See Fig 1 for information on total recruitment and reasons for exclusion after screening. Screening began with an initial phone or online screening for broad eligibility. Participants who appeared likely to qualify based on this initial screening were invited for an in-person screening consisting of a physical examination, an electrocardiogram, a modified Structured Clinical Interview for DSM-IV [33], and drug use history and health questionnaires. Inclusion criteria included consumption of at least 4 alcoholic drinks on one occasion in the last month but not more than 10 per week. Candidates were disqualified if they used any illicit drug more than once per week, or smoked more than 10 cigarettes per week. We recruited specifically for casual experience with ALC to minimize the likelihood of nausea or vomiting in response to ALC, as well as because many social drinkers also report some lifetime use of stimulants and marijuana. In the present sample, 83% of participants had previously used marijuana, and 33% had used a stimulant like AMP (see Table 1 for additional information about sample drug use). Because our interest was in subjective responses during early, non-dependent use, we excluded individuals with past lifetime dependence on ALC, marijuana, or stimulants. Candidates were also disqualified if they drank more than 3 cups of coffee per day, had a BMI outside the range of 19–26 kg/m^2^, had a medical condition requiring medication or contraindicating administration of the drugs, had DSM-IV Axis I mood, anxiety, eating, or psychotic disorder in the last year, had less than high school education, or lacked English fluency. Women were excluded if they were pregnant, nursing, or planning to become pregnant in the next three months. Women who were not on birth control were only scheduled during the follicular phase of the menstrual cycle, as hormone levels may affect AMP responses [34]. Participants completed personality questionnaires during screening (see 2.5.3).

**Table 1:** Mean (SD) age, education, and drug use of participants (n = 24).

Variable	Mean (SD) or %
Age (years)	24.6 (2.8)
Education (years)	16 (1.4)
**Past Month Substance Use**	
Caffeine	
% reporting past month use	92%
Number of servings/month in those reporting any use	54.4 (38.7)
Nicotine	
% reporting past month use	4%
Number of cigarettes/month in those reporting any use	7.5 (0)
Alcohol	
% reporting past month use	100%
Number of drinks/month in those reporting any use	17.6 (7.3)
Cannabis	
% reporting past month use	33%
Number of uses/month in those reporting any use	2.7 (2.3)
**Lifetime Substance Use**	
Cannabis	
% reporting lifetime use	83%
Number of lifetime uses in those reporting any use	141.0 (356.9)
Stimulants	
% reporting lifetime nonmedical use	33%
Number of lifetime uses in those reporting any use	3.25 (2.12)

Participant demographic information is presented in Table 1. Participants were primarily Caucasian (n = 17, 70.8%), in their 20s (M = 24.6, SD = 2.8), with a college education (M = 16years of education, SD = 1.4), and light to moderate drug use.

After screening, a total of 28 participants were randomized, with 4 participants discontinuing the study after one or more sessions, 3 due to scheduling conflicts, one due to expected side-effects of alcohol, i.e. nausea and vomiting. No other adverse events were noted. Participants who discontinued were replaced in the randomization order until a final analyzable sample of 24 was achieved, at which point the trial was stopped. A sample size of 24 was selected to provide 80% power for the correlations between drug effects observed in the most similar previous studies [15–17].

All participants provided written informed consent. All procedures were approved by the University of Chicago Institutional Review Board, and were conducted in accordance with the Declaration of Helsinki. The original approved IRB protocol is available as Supplemental Material to the current report (S2 File). All procedures were conducted in the Human Behavioral Pharmacology Laboratory at University of Chicago, an outpatient laboratory designed to resemble a comfortable “living room” environment. This study has been registered with www.clinicaltrials.gov as NCT02485158. This study was not registered before enrollment of participants because prior to PLOS One’s identification of it as a clinical trial, we did not consider it to meet the definition; participants were not randomly assigned to health-related interventions, but rather received single acute doses of abused drugs in a wholly within-subject design, no health-related outcomes were collected, and the trial was not intended to support any clinical usage or indication for ALC, AMP or THC. The authors confirm that all ongoing or related trials for these drugs that they have responsibility for are now registered.

### 2.3 Drugs, Doses and Timing

Drug doses, methods of administration, and timing of administration were selected based on previous studies conducted in our laboratory. Specifically, we attempted to select doses that were safe and effective in our typical participant population and that produced roughly equivalent changes in “Feel Drug” at peak based on previous data. We also attempted to synchronize the peak time of effect for these drugs by staggering their administration.

An AMP dose of 20mg reliably produces subjective reports of drug liking and euphoria, even in participants inexperienced with the drug. Extensive testing of this dose in our laboratory [34–37] indicates that the subjective effects of AMP peak at 90min after capsule administration, with a mean increase of approximately 30 points (SD = 26) on a 1–100 “Feel Drug” scale at that time. AMP was administered in size 00 gelatin capsules with dextrose filler.

Based on previous studies in our laboratory [38, 39], we selected a 7.5mg dose of THC. This dose was also expected to have peak subjective effects 90min after capsule administration, with a mean increase of approximately 42 points (SD = 26) on a 1–100 “Feel Drug” scale. We used THC instead of smoked whole plant marijuana for two primary reasons: 1) It is easier to control dosing. Dosing smoked whole-plant marijuana would require a paced-puffing procedure, which can be unpleasant for participants, and also still may not fully control for inhalation depth [40]. 2) It lent itself more easily to an effective placebo blind. Using smoked whole plant marijuana in a blind design would have required a complex triple dummy procedure with administration of a capsule, beverage and cigarette at every visit. Further, in previous studies, oral THC produced similar subjective effects to whole plant marijuana on relevant subjective measures [41, 42], and smoked administration of THC/marijuana was generally only slightly preferred to oral administration [43]. Thus, we decided that the ease of administration of oral THC outweighed the naturalistic advantages of smoked whole-plant marijuana. Oral THC was administered in size 00 gelatin capsules with dextrose filler, identical to those used for the AMP.

We selected the alcohol dose of 0.8g/kg for men (0.7g/kg for women, to adjust for sex differences in total body water)[44] because this dose has been safely used in many previous alcohol studies [45, 46]. Based on previous studies, we expected peak subjective effects to occur at approximately 30min after the initiation of drinking, with a mean increase of approximately 58 points (SD = 22) on a 1–100 “Feel Drug” scale. The alcohol beverage was 95% alcohol (Everclear) mixed with cranberry juice to form a 16% by volume solution. The dose was 450 ml/70 kg (i.e., adjusted for weight), divided into equal thirds (i.e., 150 ml/70 kg for each third). The beverage was served cold in opaque, lidded cups and consumed through a drinking straw. Participants had 5min to consume each third under staff supervision. Thus, the entire alcohol administration procedure was 15min long. We have used this procedure previously in several studies [45, 47].

The placebo capsules consisted of dextrose filler in size 00 gelatin capsules identical to those used for AMP and THC. The placebo beverage was cranberry juice plus 1% alcohol added as a taste mask, served in equal volume and in identical cups to the alcohol. Each drug was compared with the placebo session either immediately before or immediately after, depending on whether the random administration order started with a placebo or a drug session. Although using three placebo sessions increased the time commitment and expense of the study, use of a single placebo combined with the phenomenon of regression to the mean would have tended to produce spurious significant correlations between our Drug vs. Placebo change scores; thus, the additional sessions were deemed necessary.

Administration times for AMP, THC and alcohol were adjusted to synchronize expected peak times: capsules (AMP, THC and placebo) were given 90 min before our expected peak time point and beverages (alcohol and placebo) were given during a 15 min drinking period that commenced 30 min before our expected peak time point. Of note, we elected to adjust the alcohol dose to body weight, but not the AMP or THC dose, because analysis of previous studies conducted in our laboratory indicated that weight does not significantly affect the subjective effects of AMP or THC, provided BMI was within our established limits. Further, we expected slightly lower “Feel Drug” ratings for AMP compared to the other drugs, but elected not to increase the dose of AMP, as there was little information available on safety of higher doses in healthy, non-stimulant abusing participants.

### 2.4. Procedure

Participants first attended an orientation wherein the study procedures were explained and informed consent was obtained. Participants were instructed to fast for 2hr prior to each study session. They were also instructed to refrain from consuming alcohol or over-the-counter drug use for 24hr before and 12hr after the session and from illicit drug use for 48hr before and 24hr after each session. Compliance with these restrictions was verified using breath ALC (Alcosensor III, Intoximeters Inc.) and urine tests for commonly used drugs (ToxCup, Branan Medical Corporation). If participants failed the breath or urine tests, they were sent home, and their session was rescheduled. Female participants provided urine samples to test for pregnancy before each session (AimStrip, Germaine Laboratories). Participants were informed that they might receive a stimulant, a tranquilizer, a marijuana-like drug, alcohol, or a placebo.

After the orientation, subjects completed six study sessions, separated by at least 48hr. For each session, they arrived at 11:00am, completed breath and urine tests, and consumed a standard snack. At 11:15am, they completed baseline mood and subjective drug effect questionnaires (see 2.5.1), and at 11:30am they consumed capsules that contained AMP, THC, or placebo. At 12:00pm, participants again completed the questionnaires, and at 12:30pm, they began the 15-minute drinking period, wherein they consumed a beverage that contained either ALC or placebo. Then at 1:00pm, 2:00pm, 2:30pm, 3:00pm, 3:30pm and 4:00pm, participants completed the same questionnaires and vital signs were assessed. At 4:30pm, they completed an exit questionnaire in which they were asked to identify which drug they thought they had received in the capsule and in the beverage. Then, provided their cardiovascular measures were within the normal range and they reported no residual drug effects, they left the laboratory.

### 2.5. Measures

#### 2.5.1. Subjective Drug Effects

The primary measure of subjective drug effects was the Drug Effects Questionnaire (DEQ) [48, 49]. On the DEQ, participants rated drug effects on a visual analog scale from 1–100 (i.e. feeling, liking, and disliking the drug effect, feeling high, and wanting more of the drug). This was the measure we used to compare positive effects across drugs, as it is not specific to drug class. Participants also completed a version of the Addiction Research Center Inventory (ARCI) [50] that includes the marijuana scale [51]. The ARCI measures effects specific to drug classes, including the effects of AMP-like drugs (A scale), morphine and benzedrine like drugs (MBG scale), lysergic acid-like drugs (LSD scale), benzedrine-like drugs (BG scale), pentobarbital-chlorpromazine and ALC-like drugs (PCAG scale), and cannabis-like drugs (M scale). We used this questionnaire as a manipulation check to ensure that the drugs produced their typical drug-specific effects in this study.

#### 2.5.2. Physiological Effects

At regular intervals, we measured heart rate and blood pressure using a digital monitor (Dinamap 1846SX, Critikon, Tampa, Florida) and blood ALC concentration using an Alco-sensor III hand-held device (Intoximeters, Inc., Saint Louis, Missouri).

#### 2.5.3. Personality measures

Personality was measured using the Multidimensional Personality Questionnaire Brief Form (MPQ) [52]. The MPQ consists of 155 items that are grouped into 11 primary trait scales (Wellbeing, Social Potency, Achievement, Social Closeness, Stress Reaction, Alienation, Aggression, Control, Harm Avoidance, Traditionalism, Absorption). These trait scales are, in turn, grouped into three superfactors (Positive Emotionality, Negative Emotionality, and Constraint). For the current study, we focused on Negative Emotionality, Constraint, and Social Potency, as described in the introduction.

#### 2.5.4 Assessment of blind

During the informed consent process, participants were told they might receive a stimulant, a tranquilizer, a marijuana-like drug, alcohol, or a placebo, and further that they might receive these drugs alone, or in combination. This was done to enhance blinding and reduce expectations. At the end of each session, participants responded to two questions: “Based on its effects, what drug do you think the capsule contained?” and “Based on its effects, what drug do you think the beverage contained?” with the response options of “Stimulant”, “Sedative”, “Marijuana-like drug”, “Alcohol” and “Placebo”. For each option except Alcohol and Placebo, examples of drugs in that category were provided, to ensure participants understood the labels.

### 2.6. Data Analysis

Each drug session was compared to the placebo session closest in time (i.e. either immediately preceding or immediately following the drug session). The primary analysis aimed to determine if individual differences in subjective drug responses on the DEQ covaried across AMP, THC, and ALC.

We first conducted a series of group-level analyses of drug effects, to confirm the timing and nature of the subjective drug effects, and investigate any possible sex differences. First, we confirmed that the peak subjective effects occurred at timepoint 3, i.e., 1pm, or 90min after the capsule and 30min after the drink, as intended (see 3.1). Second, we profiled effects of the drugs *compared to placebo*, on both our primary variables of interest from the DEQ, and on the ARCI, to test whether the drugs produced their expected drug-specific effects. To do this, we calculated peak change scores for the DEQ and ARCI scales by subtracting the pre-capsule/beverage score from the score at 1pm, or timepoint 3, the peak for each session. We then subtracted the peak change score of the placebo session from the peak change score of the corresponding drug session. These Drug-Minus-Placebo peak change scores were used in 11 single-sample t-tests for each drug (one for each DEQ and ARCI scale) to determine which scales were significantly elevated by which drugs. Third, we examined the effects of the drugs *compared to each other* on both the DEQ and ARCI, to determine whether the drugs differed in their effects on these scales (for example, two drugs might both significantly elevate the DEQ Feel Scale relative to placebo, the question addressed by the previous set of t-tests, but yet differ in the magnitude of their effects on that scale). To do this we conducted 11 one-way RMANOVAs (one for each DEQ and ARCI scale) with drug (AMP, ALC, and THC) as the independent variable and Drug-Minus-Placebo peak change scores as the dependent variable. All significant main effects of drug were followed up with paired-sample t-tests comparing Drug-Minus-Placebo peak change scores for individual pairs of drugs. Together these analyses allowed us to profile how the drugs affected the primary variables of interest on the DEQ, determine whether the drugs differed in terms of the magnitude of those effects, and confirm whether the drugs produced their typical drug-specific effects on the ARCI. Last, we tested for any sex differences in drug effects on our primary variables of interest using 5 independent sample t-tests for each drug to examine whether participant sex influenced the effects of the drugs on any of the DEQ scales.

After these group-level analyses, we then conducted the primary analysis of individual differences (see 3.2). Here, we again used the Drug-Minus-Placebo peak change scores on the DEQ, as a given subject’s score on this variable represents the strength of the drug effect relative to the placebo effect for that specific individual. We carried out 15 correlations examining the relationship between AMP and THC, THC and ALC, and ALC and AMP Drug-Minus-Placebo Peak Scores for each of the DEQ scales. We also tested whether any of these relationships were moderated by participant sex using moderated multiple regression. To do this we carried out 15 regressions in which we arbitrarily designated one drug effect as the dependent variable and one as the independent variable, and included participant sex and the interaction between participant sex and the appropriate drug effect as independent variables. In the event that a significant interaction with sex was found, we conducted post-hoc correlational analyses in men and women separately to explore the effect. Further, we conducted one specific replication analysis of individual differences. Subjective response to ALC can vary substantially across individuals, and the relationship between the responses to AMP and ALC may be modulated by the degree to which participants exhibit a stimulant-like response to alcohol. In one previous report, only stimulant responders to ALC demonstrated a relationship between AMP and ALC responses on the ARCI A scale [17]. Therefore, we repeated this specific analysis, performing a median split based on the subjects’ ARCI A score in response to ALC to divide subjects into “stimulant responders” and “sedative responders,” and examining correlations between ARCI A scores in response to AMP and ALC within these groups.

Next, in a secondary analysis, we examined the drugs’ effects in relation to personality scores on the MPQ (see 3.3). To do this, we constructed 15 multiple linear regressions to determine the predictive value of the MPQ Negative Emotionality, Constraint, and Social Potency scales for Drug-Minus-Placebo peak change scores for each scale of the DEQ for each drug. Last, we conducted simple descriptive statistics to examine the success of our blinding procedure.

To ameliorate the increase in Type I Error that accompanies multiple hypothesis tests, for the initial set of analyses characterizing the group-level drug effects, we use a Bonferroni-corrected p-value for 33 comparisons of 0.002, and report as “marginal” any effects with p ≤ 0.05, except for follow-up analyses of significant omnibus effects, in which case we consider p ≤ 0.05 significant. For the primary analyses of individual differences we use a Bonferroni-corrected p-value for 15 comparisons of 0.003, and report as “marginal” any effects with p ≤ 0.05.

## Results

### 3.1 Group-Level Drug Effects

**Fig 2:**
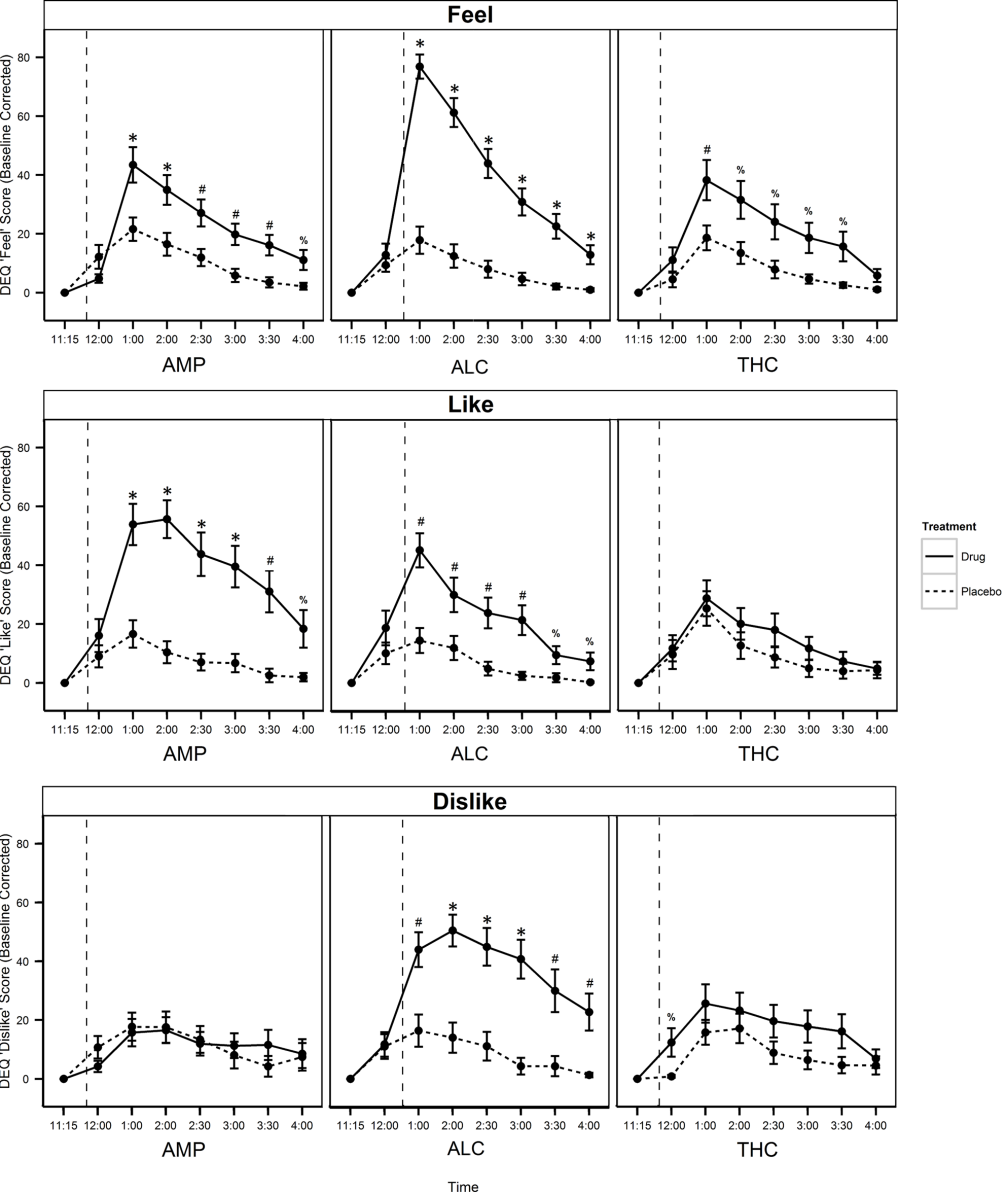
Changes from pre-capsule/drink ratings in DEQ scales. Mean changes for “Feel Drug” (top row), “Like Drug” (middle row), and “Dislike Drug” (bottom row) following AMP (left column), ALC (middle column) and THC (right column) relative to their corresponding placebos across the session. Vertical dashed lines indicate time of capsule or drink administration. The values represent the mean ± s.e.m for each timepoint. ^%^ *P*<0.05, ^#^ *P*<0.01, *P<0.001.

**Fig 3:**
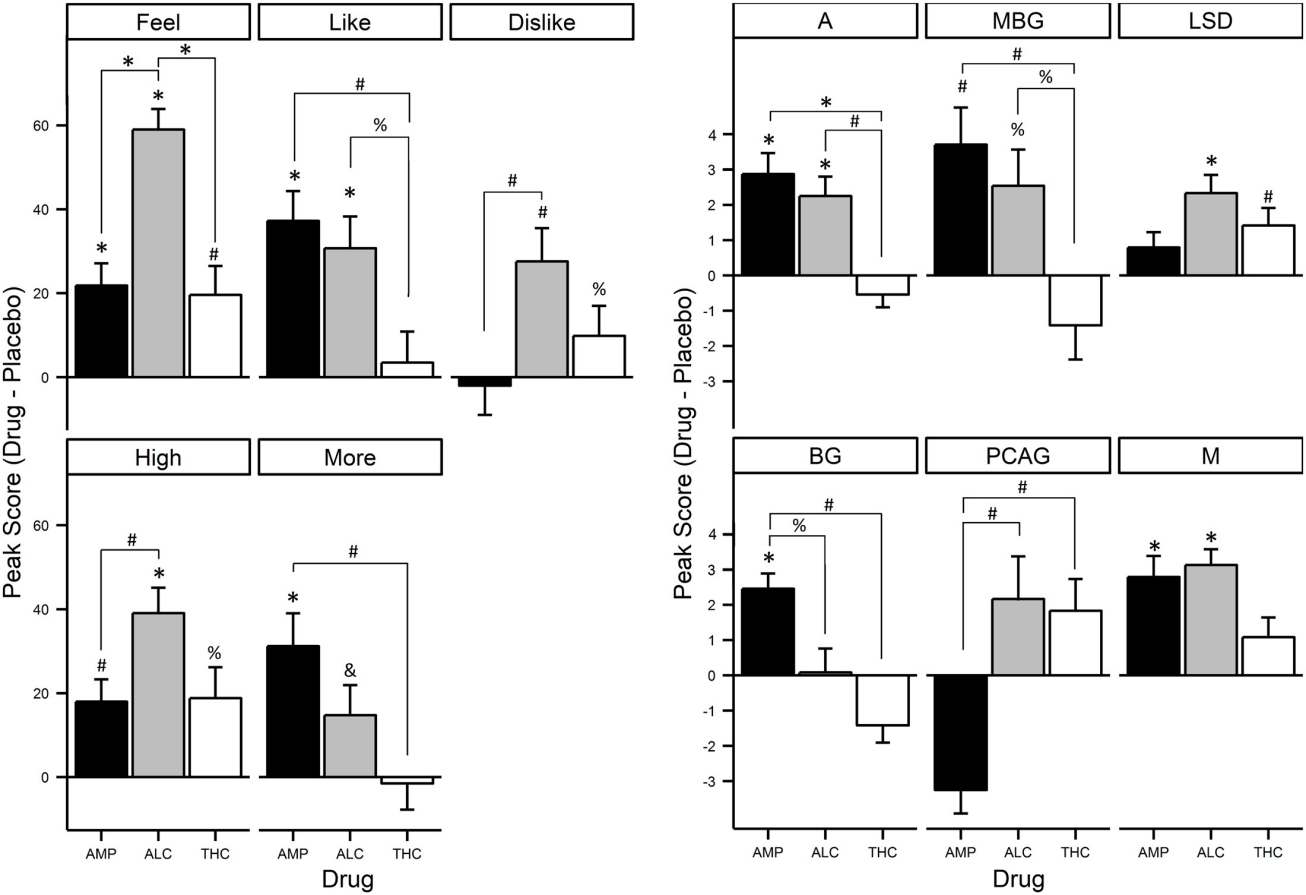
Mean peak changes from pre-capsule/drink in DEQ and ARCI scales. Left: Mean peak change from pre-capsule in DEQ “Feel,” “Like,” “Dislike,” “High,” and “Want More” scores by drug. Right: Mean peak change in ARCI A, MBG, LSD, BG, PCAG, and M scores by drug. The values represent the mean ± s.e.m at timepoint 3. ^%^ *P*<0.05, ^#^ *P*<0.01, *P<0.001, ^&^ *P* = .051.

We first confirmed that all three drugs peaked on the DEQ “Feel” scale at the planned timepoint, 1pm or timepoint 3 (Fig 2, top panel). The peak effects of AMP, ALC, and THC on each DEQ scale are presented in Fig 3 and discussed below. Means, standard deviations, and post-hoc comparisons of the magnitude of these effects are presented in S1 Table.

All three drugs elevated the “Feel” scale of the DEQ: AMP (*t*(23) = 4.107, *p* < .001) and ALC (*t*(23) = 12.069, *p* < .001) significantly and THC (*t*(23) = 2.875, *p* = .009) marginally; further, the ANOVA testing for differences in the magnitude of these effects was significant (*F*(2,22) = 20.294, *p* < .001), with post-hoc tests indicating that ALC induced a significantly greater response on “Feel Drug” than both AMP and THC; see Fig 3 and S1 Table. Subjects also reported significantly “liking” the effects of AMP (*t*(23) = 5.255, *p* < .001) and ALC (*t*(23) = 4.046, p = .001), but not THC (*t*(23) = 0.466, *p* = .65). The ANOVA testing for differences in the magnitude of these effects was marginally significant (*F*(2,22) = 5.187, *p* = .01), with post-hoc tests indicating that subjects reported equivalent responses to AMP and ALC, but liked both drugs more than THC (see Fig 3 and S1 Table). Interestingly, subjects also reported disliking the effects of ALC (*t*(23) = 3.493, *p* = .002), but not AMP(*t*(23) = -.285, *p* = .78), or the THC (*t*(23) = 1.373, *p* = .183). The ANOVA testing for differences in the magnitude of these effects was marginal (*F*(2,22) = 4.709, *p* = .02), with post-hoc tests indicating that subjects reported disliking ALC more than AMP (see Fig 3 and S1 Table). Next, all three drugs caused subjects to feel “high”: ALC (*t*(23) = 6.393, *p* < .001) significantly and AMP (*t*(23) = 3.240, *p* = .004), and THC (*t*(23) = 2.560, *p* = .02) marginally. The ANOVA testing for differences in the magnitude of these effects was also marginal (*F*(2,22) = 4.685, *p* < .02), with post-hoc tests indicating that subjects felt more “high” in response to ALC than AMP (see Fig 3 and S1 Table). Finally, subjects reported “wanting more” AMP (*t*(23) = 3.986, *p* = .001), but not ALC (*t*(23) = 2.060, *p* = .05) or THC (*t*(23) = -.240, *p* = .81). The ANOVA testing for differences in the magnitude of these effects was marginal (*F*(2,22) = 4.247, *p* = .03), with post-hoc tests indicating subjects reported wanting more AMP to a greater degree than they reported wanting more THC (see Fig 3 and S1 Table). There were only two marginal effects of sex on DEQ responses, with women reporting slightly higher “Feel” and “High” scores for THC (t(22) = 2.392, p = .03 and t(22) = 3.017, p = .006, respectively).

On the ARCI, we expected the A, PCAG, and M scales to be most strongly affected by AMP, ALC, and THC, respectively. The peak effects of AMP, ALC, and THC on each ARCI scale are presented in Fig 3, and discussed below. Means, standard deviations and post-hoc comparisons of the magnitude of these effects are presented in S2 Table.

First, on the A scale, which represents prototypical AMP effects, AMP and ALC both increased scores (*t*(23) = 4.842, *p* < .001; *t*(23) = 4.072, *p* < .001 respectively), while THC did not. Consistent with this, the effects of the three drugs were significantly different (*F*(2,22) = 13.068, *p* < .001), as AMP and ALC induced equivalent effects, while both produced stronger effects than THC (Fig 3, S2 Table). On the MBG scale, which represents euphoric experiences, AMP significantly increased scores (*t*(23) = 3.531, *p* = .002) and ALC marginally increased scores (*t*(23) = 2.480, *p* = .02), while THC did not. The ANOVA testing for differences in the magnitude of these effects was marginally significant (*F*(2,22) = 7.407, *p* = .003), such that peak scores on AMP and ALC were not significantly different, but both AMP and ALC produced greater MBG effects than THC (Fig 3, S2 Table). Third, on the LSD scale, measuring dysphoric drug experiences, ALC and THC increased scores relative to placebo (*t*(23) = 4.516, *p* < .001; *t*(23) = 2.856; *p* = .009), but AMP did not. However, the ANOVA testing for differences in the magnitude of these effects was not significant (*F*(2,22) = 2.777, *p* = .08). On the BG scale, which also contains euphoria items, AMP significantly increased scores (*t*(23) = 5.720, *p* < .001), while THC significantly reduced scores (*t*(23) = -2.899, *p* = .008). Consistent with this, the ANOVA showed that peak scores on these three scales differed significantly across the three drugs (*F*(2,22) = 12.920, *p* < .001) with AMP inducing a significantly larger effect on the BG scale than ALC and THC (Fig 3, S2 Table). On the PCAG scale, measuring sedative-like effects of ALC, ALC surprisingly did not significantly increase scores (*t*(23) = 1.798, *p* = .09) but, as expected, AMP decreased scores (*t*(23) = -4.903; *p* < .001). The ANOVA testing for differences in the magnitude of these effects was significant (*F*(2,22) = 14.186, *p* < .001), such that ALC and THC produced similar increases in sedation, while AMP reduced sedation significantly compared to both ALC and THC, (Fig 3, S2 Table). Finally, and somewhat unexpectedly, AMP and ALC, but not THC, significantly increased scores on the M scale, which measures marijuana-like effects (AMP: *t*(23) = 4.734, *p* < .001; ALC: *t*(23) = 6.981, *p* < .001; THC: *t*(23) = 1.942, *p* = .07). However, these differences were small and the ANOVA did not show a significant difference in the response to the three drugs on this scale (*F*(2,22) = 3.084, *p* < .07; Fig 3). Only two marginal sex difference were seen on the ARCI, with women reporting higher LSD scores in response to THC (t(22) = 2.639, p = .02), but lower M scores in response to THC (t(22) = 2.478, p = .02).

### 3.2. Correlations between Subjective Responses

**Fig 4:**
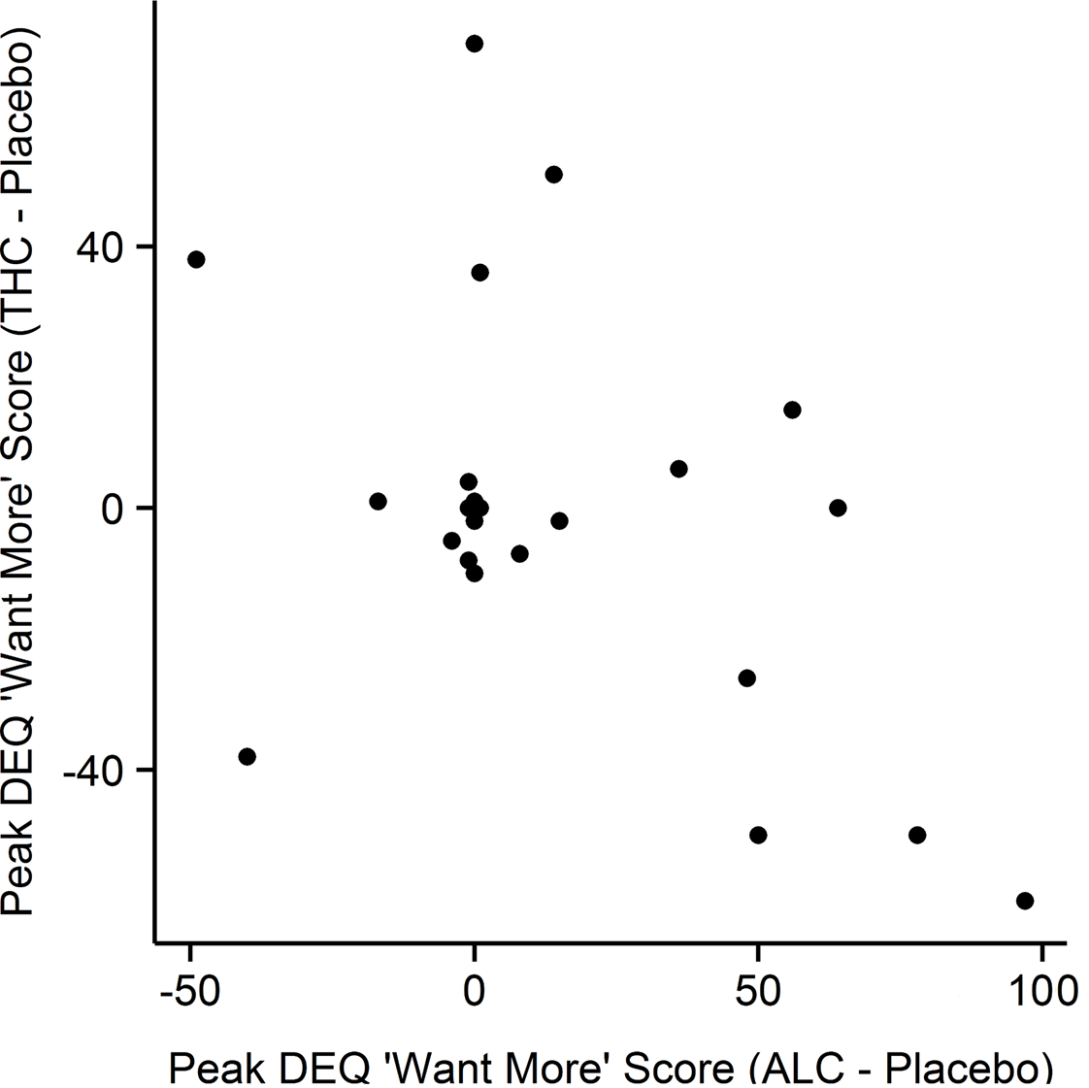
Scatter-plot of peak effects of ALC and THC on the DEQ "Want More" scale. Subjects who wanted more ALC reported wanting less THC (r = -.47, p = 0.02).

We examined the relationship between the subjective responses to the three drugs on the DEQ scales. Subjects’ responses to ALC and THC on DEQ ratings of “want more” were marginally negatively correlated (r = -.46, p = .02, Fig 4). No other significant correlations were observed. Correlations between each pair of drugs on the DEQ scales are presented in Table 2. We then further examined whether participant sex affected correlations between drug responses, using moderated regressions. Sex marginally moderated the relationship between DEQ ‘Dislike’ scores in response to ALC and THC (B = .478, *t*(23) = 2.178, *p* = .04). Post-hoc testing showed there was a marginal inverse correlation between reported “disliking” of ALC and THC in women (r = -.663, *p* = .02), but not men (r = .154, *p* = .63).

**Table 2:** Correlations between AMP, ALC, and THC Drug-Minus-Placebo change scores at timepoint 3 on Drug Effect Questionnaire (DEQ) items "Feel," "Like," "Dislike," “High,” and “Want More”.

Feel				Like			
	AMP	ALC	THC		AMP	ALC	THC
AMP				AMP			
ALC	.047			ALC	-.131		
THC	-.163	.110		THC	-.091	-.144	
Dislike				High			
	AMP	ALC	THC		AMP	ALC	THC
AMP				AMP			
ALC	.200			ALC	.222		
THC	-.271	-399		THC	-.268	-.056	
Want More							
	AMP	ALC	THC				
AMP							
ALC	.135						
THC	-.245	-.464*					

*p< 0.05

Our specific replication analysis that divided subjects into “stimulant responders” and “sedative responders,” and examined correlations between ARCI A scores in response to AMP and ALC within these groups found no relationship between ARCI A scores to AMP and ALC in sedative responders (r = .008, p = .98), but a marginally significant relationship in stimulant responders (r = .601, p = .05), consistent with previous results. However, using a Fisher r-to-z transformation, we did not see a significant difference between these two correlation coefficients (z = -1.46, p = 0.14).

### 3.3. MPQ and Drug Effects

Three personality trait predictor variables (MPQ social potency, negative emotionality, and constraint) were entered into a multiple linear regression model to predict subjective responses to each drug on each DEQ scale. All of these multiple regressions are shown in S3 Table. Only one regression was marginally significant. The personality variables predicted 32% of the variance in DEQ “like” score in response to ALC, (F(3,20) = 3.08 *p* = 0.05). MPQ Constraint was the only significant individual predictor, with lower levels of Constraint predicting higher DEQ “like” scores (B = -3.215, t(20) = 2.745, *p* = .012).

### 3.4. Assessment of Blind

Results of the exit questionnaire assessing the blind are presented in Table 3. Because participants identified the capsule and the beverage separately, for each drug session and paired placebo session we present: 1) The number of participants who identified the capsule as containing the type of drug actually administered in the drug session 2) The number of participants who identified the beverage as containing the type of drug actually administered in the drug session (note—for both AMP and THC a large number of individuals identified the beverage as containing the drug, even though it was actually administered in the capsule) 3) The number of participants identifying the type of drug actually administered at all (this includes participants who identified the type of drug actually administered and some other active drug) 4) The number of participants identifying only the type of drug actually administered (i.e. they identified no other active drugs) and 5) The number of participants identifying both the capsule and the beverage as placebo.

**Table 3:** Results of exit questionnaire assessing blinding.

Drug/Identification	Active Drug Session	Matched Placebo Session
**Amphetamine**		
Capsule Identified as Stimulant	45.8%	0%
Beverage Identified as Stimulant	41.7%	12.5%
Total Identifying a Stimulant	70.8%	12.5%
Total Identifying Only a Stimulant	45.8%	45.8%
Total Identifying Only a Placebo	0%	50%
**Alcohol**		
Capsule Identified as Alcohol	4.2%	4.2%
Beverage Identified as Alcohol	87.5%	4.2%
Total Identifying Alcohol	91.7%	4.2%
Total Identifying Only Alcohol	62.5%	4.2%
Total Identifying Only a Placebo	0%	41.7%
**THC**		
Capsule Identified as Marijuana-like Drug	8.3%	12.5%
Beverage Identified as Marijuana-like Drug	37.5%	4.2%
Total Identifying a Marijuana-like Drug	41.7%	16.7%
Total Identifying Only a Marijuana-like Drug	33%	16.7%
Total Identifying Only a Placebo	16.7%	33.3%

As can be seen from Table 3, the success of the blind was mixed. It was most effective for THC, with only 41.7% of participants identifying a marijuana-like drug at all, and least effective for ALC, with 91.7% of participants identifying alcohol.

## Discussion

In this study, we aimed to determine if healthy young adults would show consistent individual differences in their responses to several drugs of abuse that are commonly co-abused. More specifically, we examined the subjective response to AMP, ALC, and THC on a measure of general drug effects (DEQ). Regarding group-level effects of the drugs, all three drugs produced significant subjective effects. However, our expectations that the A, PCAG, and M scales on a drug-specific measure (the ARCI) would uniquely reflect the effects of AMP, ALC, and THC, respectively, were only partially confirmed. AMP increased scores on the A scale, but ALC did not increase scores on the PCAG scale, nor did THC increase scores on the M scale, despite significantly affecting other scales such as “Feel Drug” and “Feel High”. In the case of alcohol, this may be because the PCAG scale measures alcohol-related sedation, which is generally more prominently on the descending limb of the blood alcohol curve, while our peak timepoint, at which all measures were taken, was on the ascending limb [53]. In many cases, a drug appeared to act strongly on a “prototypical” scale for a different drug, suggesting more overlap in subjective drug effects than expected. For example, although THC did not induce a significant response on the M scale, AMP and ALC did. Similarly, ALC induced a response on the A scale that was equivalent in magnitude to AMP. Comparatively few sex differences were seen in group-level subjective effects. Those that were evident primarily affected responses to THC, with women reporting marginally stronger and less pleasant responses to THC. Yet although all three drugs produced significant group-level subjective effects, with a great deal of overlap in the nature of these effects, individual subjects’ responses to the three drugs were largely unrelated. This was contrary to our primary hypothesis. Indeed, the only relationship seen in the overall sample was a marginal and negative one, between DEQ “want more” scores in response to ALC and THC. We also found a marginal negative relationship between “disliking” of ALC and THC, but in women only. We did find suggestions of a positive relationship in individuals who showed certain drug effects, as we replicated a previous finding that ARCI A responses to ALC and AMP were correlated only in those individuals who showed a stimulant response to ALC. However, it should be noted that although this correlation was only significant in stimulant responders, when we directly compared the correlations in stimulant and sedative responders, they were not significantly different. This may be due to the small sample size (n = 12) once the groups were sub-divided, and suggests this finding should be subject to further replication. In our exploration of the influence of personality in the subjective response to drugs, one personality factor marginally predicted subjective responses to ALC, but none predicted responses to more than one drug.

Our finding that the subjective effects of AMP, ALC, and THC were either unrelated or possibly negatively related in healthy individuals suggests that the general version of our hypothesis, i.e. that certain individuals will show strong positive subjective responses across classes of co-abused drugs, is not supported. Instead it appears that individuals who have certain specific responses to a drug (e.g. stimulant responses to alcohol) may show correlated responses to other drugs that produce similar effects (e.g. stimulants). This is suggested by our replication of a previous finding that stimulant responses to ALC and AMP were correlated only in individuals who showed stimulant responses to AMP. This is also consistent with previous research showing that sedative-like subjective responses to ALC were positively correlated with sedative responses to benzodiazepine and diazepam [15], and that responses to caffeine and nicotine, two stimulants, are positively correlated [18]. Taken together with our findings, this suggests that relationships between drug effects may be evident only when the drugs in question elicit qualitatively similar subjective responses. This is more consistent with a “drug of choice”, or “effect of choice” model, in which individuals prefer a particular drug or drug class and dislike others, possibly based on personality or other individual differences. This is supported by our findings that wanting more ALC was inversely related to wanting more THC, and by our results suggesting that personality characteristics predicted responses to specific drugs, but not more than one drug.

Regarding our exploratory analysis with personality, we found support for one previously reported relationship between specific traits and specific drug effects, but not others. Our finding that constraint, or a lack of impulsivity, predicts a lower ALC-induced Liking is consistent with previous findings that trait impulsivity is associated with a greater stimulant response to ALC [28]. However, despite expectations based on previous findings, we found no relationship between personality and response to AMP or THC. We have previously found that the traits of social potency and impulsivity were related to AMP-induced euphoria [27], but the present sample was much smaller (24 compared to 286), raising question about power. Others have reported that negative emotionality predicts problematic drinking [30], but negative emotionality was not associated with subjective response to acute doses of ALC in our sample. It should be noted that we used measures of personality and of drug effects that were different than some of the measures used in other studies. Although it is believed that there is significant overlap in the constructs measured across personality instruments [52], it is possible that these results might have differed if an alternate personality scale or outcome measures were employed.

We were surprised at the lack of a relationship between certain drug effects in our study, as previous literature suggested they might be related. One possibility is that these inconsistencies may be explained by methodological factors. For example, there have been reports that subjective responses to ALC and marijuana are related [19, 54]. However, these studies used retrospective reports of users’ prior experiences, which may be more subject to reporting bias. Further, these studies assessed response to smoked marijuana (the most typical route of use outside the laboratory), rather than oral THC, which could yield different subjective effects [55, 56], although c.f. [43]. In addition, these studies examined responses in both community and clinical samples of users of ALC, marijuana, and tobacco, which may have been weighted towards more frequent users of drugs. Responses to drugs in frequent users may be influenced by uncontrolled factors related to their past experience with drugs, and thus may not reflect responses during early use, which were the focus here.

There were several limitations of the current study. First, most of the effects we observed were marginal, and given the small sample size, should be considered tentative and in need of replication. Second, all three drugs did produce significant subjective responses, but evident dosing differences may have masked relationships between subjective effects. Specifically, despite attempts to match the timing and intensity of the drug experiences, subjects reported significantly higher ratings of “feel drug” in response to ALC compared to the other drugs. Future studies should adjust dosing, or consider administering multiple doses of each drug. Third, our dose of oral THC failed to elevate the ARCI M scale, or produce overall positive subjective effects. It should be noted that this does not mean that our participants universally disliked the THC dose. Indeed, approximately half of participants reported liking THC more than placebo. Rather, it appears to indicate that the subjective effects of oral THC were more variable than those of our other drugs. Consistent with this, THC was also the only drug to show possible sex differences in overall effects. These sex differences were not wholly consistent with the literature, as our female participants reported stronger but more unpleasant effects of THC, while in the best powered study to date to examine this question, women reported stronger and more pleasant effects of marijuana [57], consistent with epidemiological evidence suggesting accelerated progression to cannabis disorders in women [58]. However, as will be noted below, our study included a population with much lighter and more variable use of marijuana than that examined in previous studies of sex differences, which may contribute to this difference. The unexpected variability in effects of THC in our study may also be related to the mode of administration (oral consumption vs. inhalation), expectancies, or the chemical composition of marijuana vs. THC [59], although it should be noted that studies suggest a rough equivalence between effects of oral THC and smoked marijuana [41–43]. These findings may also represent that THC does not produce strong positive effects in many individuals in early use, with greater positive effects emerging over time, and our sample contained fewer regular marijuana users [60]. Taken together, our findings suggest that investigators comparing responses to marijuana with responses to other drugs should use smoked whole-plant marijuana despite the greater difficulties with administration and blinding, and recruit for more regular use of marijuana. Related to this previous point, although recruiting “social drinkers” typically produces a population with light but reasonably varied recreational drug use, which was our goal, it should also be noted that our participants may already have selected ALC as their “drug of choice”, given their more frequent use of it compared to THC and AMP. Such a pre-existing preference might also have masked relationships between drug effects. This is particularly true in light of the fact that the blind was not wholly successful. Although only partial blinding is typical in acute administration studies that administer subjectively detectable amounts of drugs with which participants have had previous experience, the partial nature of the blind increases the possibility that subjective responses to the drug could be influenced by expectations and pre-existing preferences. Although it would be very difficult from a practical and ethical standpoint to perform a study such as this one with a wholly drug naïve sample, future studies should attempt to recruit a population that is more evenly matched on use of all three drugs to reduce the influence of possible pre-existing preferences. Last, as with all studies that utilize volunteer participants, it must be noted that individuals who volunteer for such studies may not be representative of the larger population, limiting generalizability.

## Conclusion

In summary, this study examined responses to AMP, ALC, and THC, three pharmacologically distinct drugs of abuse, in the same individuals. These drugs act via both discrete and shared brain circuitry. The results did not support our hypothesis that there would be commonalities in subjective responses across these commonly co-abused drugs. On the contrary, we found that the same individuals responded oppositely to ALC and THC on two scales. In addition, our exploratory analysis indicated that some drug responses were related to personality traits, but there were no personality traits that predicted responses across multiple drug types. However, we did find that individuals who responded in a qualitatively similar way across two drug types (e.g. stimulation in response to AMP and ALC), also showed relationships between their responses to those drug types, suggesting that relationships between the response to two drugs of different classes may only exist if these two classes of drugs share similar subjective effects. These findings do not support the idea that drugs from different drug classes produce common subjective responses in certain individuals, but rather incline towards a “drug of choice” model, in which individuals may be susceptible to particular drug classes based on personality or other individual differences.

## Supporting Information

S1 File CONSORT checklist documenting reporting criteria for a clinical trial. (DOC)

S2 File Original protocol as approved by the University of Chicago IRB, per reporting criteria for clinical trials. (DOCX)

S1 Table Post-hoc t-tests comparing peak drug effects relative to placebo for each pair of drugs (AMP vs. ALC, AMP vs. THC, ALC vs. THC) on the DEQ "Feel", "Like", "Dislike," and "Want More" scales. (DOCX)

S2 Table Post-hoc t-tests comparing peak drug effects relative to placebo for each pair of drugs (AMP vs. ALC, AMP vs. THC, ALC vs. THC) on the ARCI A, MBG, LSD, BG, PCAG, and M scales. (DOCX)

S3 Table Linear regression model of three MPQ scales (Negative Emotionality, Constraint, and Social Potency) and their contribution to the effects of AMP, ALC, and THC on the DEQ. (DOCX)
