# Supplementary material for: A Preliminary Investigation of Individual Differences in Subjective Responses to D-Amphetamine, Alcohol, and Delta-9-Tetrahydrocannabinol Using a Within-Subjects Randomized Trial
Source: PLoS One. 2015 Oct 29;10(10):e0140501. doi: 10.1371/journal.pone.0140501 (PMC4626040; doi:10.1371/journal.pone.0140501)
Supplement: S2 File — (DOCX) [file pone.0140501.s002.docx]

**Detailed Protocol Narrative**

**Title:** Individual differences in responses to drugs

**PI:** Dr. Harriet de Wit

**Co-Investigators:** Dr. Margaret Wardle, Dr. Emma Childs, Dr. Royce Lee

**Version Date:** 5/17/13

**Objectives:**  **To examine whether individual differences in acute responses to drugs co-vary across three drugs from different drug classes: alcohol, amphetamine and delta-9-tetrahydrocannabinol (THC). We will determine whether individuals who experience greater rewarding effects from one drug also respond experience more rewarding effects from other types of drugs.**

**Aim 1.** Examine correlations across the three drugs for the rewarding subjective effects of drugs, such as feeling euphoric, and “high”.

**Aim 2.** Examine correlations across the three drugs for a behavioral economic index of demand for drugs, the Multiple Choice Procedure.

**Aim 3.** Examine correlations across the three drugs for drug effects on emotional responses to external stimuli, such as enhanced pleasure responses to positive stimuli and dampened negative responses to stressful stimuli.

**Aim 4.** Examine correlations across the three drugs for the drugs’ effects on impulsivity.

**Aim 5**. Examine correlations across the three drugs on cardiovascular measures, such as heart rate and blood pressure.

**Background:** Abuse of more than one drug (‘polydrug use’) is extremely common. Most drug users misuse multiple drugs, and they commonly develop dependence on more than one drug (Kedia et al. 2007). This raises the possibility that a common underlying factor influences their reactions to a range of drugs. It has been well documented that individuals vary in their acute responses to individual drugs of abuse (e.g. Alessi et al. 2003b; de Wit et al. 1986; Holdstock & de Wit 2001; Sher & Walitzer 1986; White et al. 2006). Some individuals experience stronger or more pleasant effects from a particular drug, while others experience aversive or weaker effects. These individual differences in acute drug responses are thought to contribute to repeated use and abuse, that is, individuals with positive effects are more likely to continue to use a given drug, while aversive effects are protective against use and abuse (de Wit & Phillips 2012). These individual differences in drug effects are also thought to have a genetic component (Hart et al. 2012a; Hart et al. 2012b). Thus, differences in acute response may be part of what puts some people genetically at risk for drug abuse and addiction. What is not known is whether individual differences in acute drug effects co-vary across drugs of abuse, i.e. whether individuals who react more positively or strongly to one drug also react more positively or strongly to other drugs of abuse. This information is critical to understanding vulnerability to addiction, and in particular, establishing whether there are general underlying vulnerabilities to all addictive drugs vs. drug-specific mechanisms (Badiani et al. 2011).

There is evidence that common factors underlie individuals’ acute responses to different drug classes. A large community sample examining self-reports of subjective drug experiences found significant correlations between reported experiences with alcohol, tobacco and marijuana, and found that these commonalities were partially genetic (Haberstick et al. 2011; Zeiger et al. 2012). However, self-reports of drug experiences occurring in natural environment are subject to reporting bias, social setting, dose differences and other confounding factors that may artificially increase or decrease correlations. Examining acute responses to drugs in a laboratory setting provides better control over these factors. Several controlled laboratory studies of individual differences in cross-drug responses also suggest shared variation in responses to multiple drugs. We have demonstrated that healthy adults who experience subjective stimulant responses to alcohol also experience greater stimulation after *d*-amphetamine, and individuals who experience sedative responses to ethanol also experience greater sedative effects of the sedative triazolam (Holdstock & de Wit 1999; Holdstock & de Wit 2001). Further, subjective liking and subsequent choice for ethanol and the sedative diazepam were correlated in healthy adults (de Wit & Doty 1994). Other researchers have replicated our results with alcohol and d-amphetamine (Stoops et al. 2003), and have also established correlations between subjective arousal responses to nicotine/caffeine and nicotine/alcohol in smokers (Perkins et al. 2001). Together this small body of controlled studies suggests that there is shared variability in acute responses across multiple classes of drugs in humans.

To date, research on commonalities in drug effects has focused on a limited outcome measures, mainly subjective responses to drugs. Although subjective responses do contribute to future use and abuse (de Wit & Phillips 2012), other acute drug responses may also predict continued drug use. One example is an objective demand for the drug, measured in terms of how much someone would pay for subsequent doses of the drug (Griffiths et al. 1993; Griffiths et al. 1996). This measure of demand appears partially independent of subjective responses to the drug (Griffiths et al. 2003). Further, we have demonstrated that drugs alter responses to external stimuli in ways that are rewarding and could encourage future use. For example, we found that amphetamine increases responses to external pleasant stimuli that are encountered under the influence (Wardle & de Wit 2012). Importantly, this was independent of the subjective euphoric effects of the drug, suggesting a distinct rewarding effect. Last, acute effects of drugs on impulsivity may contribute to further use. Although acute increases in impulsivity during use may not be “rewarding” effect, it may contribute to loss of control over use and binging. Thus, in this study we intend to examine a more comprehensive set of acute drug effects, including behavioral as well as subjective effects, to identify cross-drug commonalities that may contribute to risk for polysubstance use and abuse.

In a six-session within-subject design with healthy adults we will examine the effects of three abused drugs: alcohol, d-amphetamine (an abused prescription stimulant), and THC (the psychoactive constituent of the abused drug marijuana). We have chosen these drugs because they have different mechanisms of primary action, via dopaminergic, GABAergic and endocannabinoid systems (McKim & Hancock 2003). Yet each has also been demonstrated to ultimately impact the mesolimbic dopamine system, a hypothesized common neural substrate for the effects of all drugs of abuse (Nestler 2005). Thus, these drugs provide a strong test of shared vs. unique factors in acute response. Because this is a preliminary pilot study intended to support development of a renewal grant application, we are limited to examining only one dose of each drug, but we have carefully selected doses based on previous experience to produce roughly equivalent effects. Each dose will be compared to a unique placebo session, giving a six-session design. At each session we will examine: 1. Subjective effects, i.e. feeling euphoric, feeling high, 2. Objective demand for drugs, i.e. amount of money participants would be willing to pay for another dose 3. Effects on emotional responses to external stimuli, i.e. positive responses to pleasant stimuli and negative responses to aversive stimuli, 4. Effects on impulsivity, 5. Cardiovascular effects, i.e. heart rate and blood pressure. Our primary hypothesis is that we will see moderate correlations across the three drugs within each type of acute drug effect, indicating that individual differences in these responses are consistent across drugs. We will also examine correlations between our various types of acute drug effects on an exploratory basis.

**Methods**

**Design:** The study will use a 6-session within-subjects double-blind design in which participants will receive 0.8 g/kg alcohol, 20mg *d*-amphetamine, and 7.5mg THC at one session each, interspersed with three placebo sessions. Each placebo session will be paired with the closest drug session to serve as its unique control condition. Both the order of the placebo/drug alternation (i.e. whether individuals receive placebo or drug first) and the order in which the drugs are administered will be randomized and counterbalanced. Drug and placebo administration will be double blind. For blinding purposes, subjects will receive both a capsule and beverage at each session, although only one drug will be administered per drug session. Each session will be 5.5 hours long, regardless of drug condition. An example condition order is shown in the table below:

*Table 1.* Example of a condition order for one participant

| Session | Capsule | Beverage |
| --- | --- | --- |
| 1. Amphetamine Session | 20mg d-amphetamine | Placebo |
| 2. Placebo Control for Amphetamine Session | Placebo | Placebo |
| 3. THC Session | 7.5mg THC | Placebo |
| 4. Placebo Control for THC Session | Placebo | Placebo |
| 5. Alcohol Session | Placebo | 8 g/kg alcohol |
| 6. Placebo Control for Alcohol Session | Placebo | Placebo |

**Participant Recruitment and Screening:** Participants will be 24 (N = 12 female, N = 12 male) healthy normal adults, ages 21 to 35. Based on previous rates of completion for six-session drug studies in our lab, to complete 24 subjects we will need to enroll 30 participants. All participants will be recruited without regard to race, religion or ethnicity through posters, advertisements and word-of-mouth. Candidates will be screened in accordance with our general screening protocol, approved by the IRB under Protocol #13681B, which includes a physical, EKG, psychiatric interview and detailed drug use history questionnaire. Because of the drugs that will be administered, the following populations are excluded for safety reasons: Individuals with a medical condition contraindicating study participation, as determined by our physician, individuals regularly using any contraindicated medications, individuals with current dependence on any drug or past dependence on alcohol, marijuana or stimulants, individuals with a past year DSM-IV Axis I mood, anxiety, eating, or psychotic disorder, women who are pregnant, nursing, or planning to become pregnant in the next 3 months. Because the self-report questionnaires we use require English fluency, we require a high school education and exclude those not fluent in English. We also restrict several demographic variables to minimize variability. We restrict the range of allowable BMIs to 19 – 26. In a previous large study using 20mg *d*-amphetamine (N = 400), within this range there was no significant correlation between psychophysiological or subjective responses to the drug and BMI (unpublished data). We will restrict the range of current drug use, as heavier users may respond differently to many abused drugs compared to light users (e.g. de Wit & Doty 1994). Participants must report having had at least 4 alcohol-containing drinks on one occasion in the past month, but individuals who consume more than 10 drinks in a typical week will be excluded. We will also exclude individuals who currently use i) any illicit drug weekly or more frequently, ii) stimulant prescription drugs, iii) more than 10 cigarettes per week, and iv) more than 3 cups of coffee per day. Women not on hormonal birth control will be scheduled only in the follicular phase of the menstrual cycle, as hormonal fluctuations may change amphetamine responses (White et al. 2002).

**Drugs and Doses:**

Drug Selection: For this preliminary study we have selected single doses of alcohol, amphetamine and THC. We selected these three drugs to provide a strong test of shared vs. unique factors in acute response. Each of these drugs has a different primary mode of action. Alcohol acts on GABA_A_ and the NMDA system, in addition to potentiating several other neurotransmitter systems (McKim & Hancock 2003). Amphetamine increases concentrations of dopamine in the synaptic cleft by increasing dopamine release and reversing the dopamine reuptake transporter, although it also has prominent effects on norepinephrine and serotonin. THC acts as a partial agonist at the CB_1_ receptor of the endocannabinoid system. Yet despite these differences in primary sites of action, all of these drugs ultimately impact mesolimbic dopamine functioning, a proposed neural substrate for the addictive effects of all drugs of abuse (Nestler 2005). In addition to common and divergent neurochemical actions, these drugs also have common and divergent acute effects. All three drugs increase ratings of liking and euphoria, and objective demand over placebo. However, they vary in their typical effects on emotional responses, with amphetamine increasing responses to pleasant stimuli, alcohol blunting responses to all emotional stimuli, and THC having no effect on responses (Ballard et al. 2012; Stritzke et al. 1995; Wardle & de Wit 2012). They also have varying effects on impulsivity, with alcohol increasing impulsivity while amphetamine decreases it. Thus, we have selected drugs with both convergent and divergent effects for a strong test of common vs. distinct underlying mechanisms. It is also important to remember that there is individual variation in each of these responses, so simply because amphetamine decreases impulsivity *on average* does not mean that it does not increase impulsivity for some individuals. A strength of the current design is that we are able to examine relationships between these effects within-subjects.

Dose Selection: It is not possible to match the drug doses exactly, because their effects are qualitatively different. However, we have selected doses that produce moderate peak effect scores on the subjective scale “Feel Drug Effect” and have carefully matched peak effect times. Figure 1 shows projected average “Feel Drug” scores for our selected doses on a scale from 0 – 100 across the study, along with expected BAL in the alcohol condition, multiplied by 1,000 to put it on the same scale. We base these projections on previous data from our lab (Ballard et al. 2012; Kirkpatrick et al., under review; Hart et al. 2012b).

*Figure 1.* Projected average “Feel Drug” scores based on previous data, along with BAL (multiplied x 1,000). Projections based on previous data from our lab using these same drugs and doses.


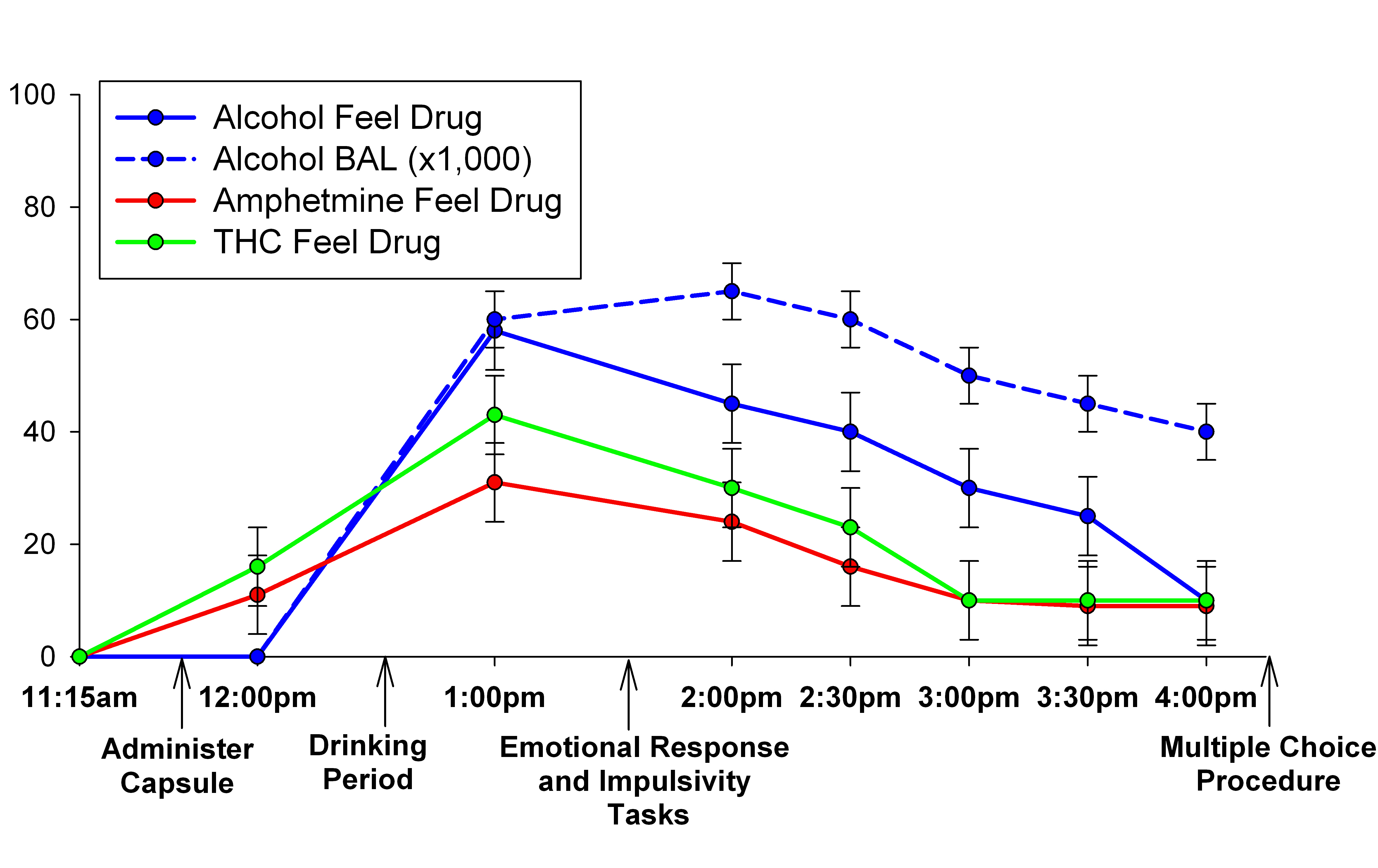


Drug Administration:

*1. Alcohol (0.8g/kg for men, 0.7g/kg for women):* Alcohol will be given in a single dose of 0.8 g/kg of body weight, which is equivalent to 4 standard drinks [a standard drink is defined as one 12 oz beer, one 5 oz glass of wine, or one 1.5 oz shot of 80 proof alcohol]. Adjustments will be made for women to receive an approximate 85% dose for that of men due to differences in total body water affecting blood alcohol concentrations (Frezza et al. 1990; Sutker et al. 1983). Oral ethyl alcohol dose will be calculated based on the estimated total body water (TBW) of each participant. The following regression equation will be used: TBW = -2.097 + 0.1069 (height in cm) + 0.2466 (weight in kg) for women and TBW = 2.447 – 0.0956 (age in years) + 0.1074 (height in cm) + 0.3362 (weight in kg) for men (Watson et al. 1981). The alcoholic drink will be 95% alcohol in a 16% solution by volume with cranberry juice cocktail. The placebo beverage will consist of the cranberry juice plus 1% alcohol added as a taste mask. Beverages will be prepared by the investigator in a volume of 450 ml/70 kg (i.e., adjusted for participant weight) and will be divided into equal thirds (i.e., 150 ml/70 kg for each third). Beverages will be served cold in opaque, lidded cups and will be consumed through a drinking straw. Research assistants will be blinded to the drug condition. Participants will have 5 min to consume each third under staff supervision. Thus, the entire alcohol administration procedure will be 15 min. We have used this procedure previously in several studies in the lab with no adverse effects (e.g. Doty & de Wit 1995; Kirkpatrick, under review). All participants must report consuming at least 4 drinks in one sitting within the previous month during screening, to ensure that they are comfortable with the selected dose of alcohol. See **Risks** section for complete safety information on alchohol.

*2. d-Amphetamine (20mg):* We will administer 20mg d-amphetamine (Dexedrine) in size 00 gelatin capsules with dextrose filler. Placebo capsules will consist of only dextrose filler. We have tested this dose extensively in previous studies (e.g. Brauer & de Wit 1995; Childs & de Wit 2009; de Wit et al. 1997; de Wit et al. 2002; de Wit et al. 1986; Hart et al. 2012b). It reliably produces subjective effects (e.g., increased ratings of feel drug, like drug), and behavioral effects (e.g., improved psychomotor performance). In the carefully screened healthy participants in our studies, we have not observed any adverse effects over many years of research with this drug. In addition doses of up to 60mg are routinely administered to adults and children for the treatment of ADHD with no associated adverse consequences (per Dexedrine Package Insert). See **Risks** section for complete safety information on amphetamine.

*3. THC (7.5mg):* We will administer 7.5mg THC (Marinol) in in size 00 gelatin capsules with dextrose filler. Placebo capsules will consist of only dextrose filler. We have used this dose and higher doses up to 15mg in several prior studies with carefully screened healthy participants with no adverse effects. We selected this dose for its rough equivalency on “Feel Drug” to 20mg amphetamine. Marinol is used clinically to aid appetite in AIDS patients and individuals undergoing chemotherapy, with doses up to 10mg being well tolerated (per Marinol Package Insert). Further, a typical “joint” of marijuana delivers between 5-25mg THC (Curran et al. 2002). Thus this dose is both in the recreational range, and previously demonstrated to be well-tolerated in both laboratory and clinical studies. See **Risks** section for complete safety information on THC.

**Procedure**

Orientation: Participants who meet criteria will first be scheduled for an orientation session. During this session, subjects will be informed that the capsules and beverages used in the study may contain a placebo, a stimulant drug (e.g. *d*-amphetamine, Ritalin), a sedative drug (e.g. diazepam, Valium), alcohol, or a marijuana-like drug (e.g. THC, Marinol). In previous studies we have found this procedure reduces expectancy effects. To further enhance blinding, participants will be told that they might receive no drugs, different doses of a drug, or one or more drugs from the list at each session. Participants will be given an oral description of the study procedures and the written consent form. After the experimenter reviews this information and the consent form with the subject, and answers any questions he/she may have, subjects will answer questions confirming their understanding of the study, and sign the informed consent document. The subject will then practice completing the tasks and questionnaires to be used in the study.

Study Session: Please see below for a full timeline of the study session. Participants will be asked to fast for 2 hours prior to study sessions, and to refrain from using alcohol or over-the-counter drugs not previously cleared by our study physician for 24 hours before and 12 hours after the session, and from illicit drugs for 48 hours before and 24 hours after each session. On study session days, participants will arrive at 11am, and consume a standardized snack. Participants will then complete a urine screening for recent drug use (ToxCup Drug Screen Cup, Brannan, Irvine CA) a screening of breath alcohol level (BAL) for recent alcohol use (Alco-sensor III, Intoximeters, St. Louis MO), and a pregnancy test (for women). Subjects who test positive for drugs will be rescheduled, and women who are pregnant will be dropped. After drug compliance testing we will obtain baseline measures of subjective mood, drug effects, and cardiovascular variables. These measures, along with BAL will taken periodically throughout the study (see below). At 11:30am, participants will ingest a capsule containing amphetamine, THC or placebo. They will be allowed to relax and watch a movie or read a book for 30 min until the drugs are absorbed, but will not be allowed to do work. At 12:00pm we will reassess BAL, subjective and cardiovascular effects. At 12:30pm subjects will consume a beverage containing 0.8g/kg alcohol or an equivalent volume of placebo in three equal portions. The portions will be administered every 5min, for a 15min total drink period. Participants will rinse their mouths out with water at the end of the drink period to ensure accurate BAL assessment. At 1pm, 15min after the end of the drink period, we will reassess BAL, subjective and cardiovascular effects. The emotional response and impulsivity tasks will begin immediately after this, and will last for approximately 45min, to coincide with the peak effect of the drugs. Tasks measuring emotional responses and impulsivity (see below) will be presented in counterbalanced order. Participants will complete subjective questionnaires every half-hour. Just before the end of the session at 4 pm they will complete the Multiple Choice Procedure, assessing how much money they would be willing to pay for the drug they had that day. Participants will be permitted to leave at 4:30 pm if they meet all of the following criteria: 1. They have a BAL less than 0.04 2. Their cardiovascular measures have returned to baseline 3. They report minimal residual drug effect. We expect most participants will be cleared to leave at 4:30pm, but will ask participants to stay longer if they do not meet these criteria, or if they wish to stay longer. In the event that participants are retained in the lab past 6pm, they will be provided with a door-to-door cab ride home. Sessions will be separated by at least 72 hours. Study drugs are expected to have cleared by the following session, with the potential exception of THC, which remains detectable in urine over a longer period of time due to its lipid solubility. However, positive THC tests following a THC session may be permitted, if the subject passes a further test of recent use, using a measure of salivary THC (Oratect III, Brannan, Irvine CA).

Timeline

11:00am – Arrival, snack, breath and urine compliance tests

11:15am – Mood (POMS), drug effect (ARCI, DEQ), cardiovascular measures

11:30am – Capsule administered

12:00pm ­­– BAL, Mood (POMS), drug effect (ARCI, DEQ), cardiovascular measures

12:30 - 12:45 pm – Drinking period

1:00pm – BAL, Mood (POMS), drug effect (ARCI, DEQ), cardiovascular measures

1:15pm – Emotional response (IAPS, DEIT) and Impulsivity (Go/No Go)

2:00pm – BAL, Mood (POMS), drug effect (ARCI, DEQ), cardiovascular measures 2:30pm – BAL, Mood (POMS), drug effect (ARCI, DEQ), cardiovascular measures 3:00pm – BAL, Mood (POMS), drug effect (ARCI, DEQ), cardiovascular measures

3:30pm – BAL, Mood (POMS), drug effect (ARCI, DEQ), cardiovascular measures

4:00pm – BAL, Mood (POMS), drug effect (ARCI, DEQ), cardiovascular measures

4:15pm – Multiple Choice Procedure

4:30pm – Cleared to leave laboratory

Debriefing: After completing the six sessions, participants will return to the lab for a final session at which they will be fully debriefed with regard to the study hypotheses, methods and the types of drugs that they received, and will be given a chance to ask any remaining questions.

**Tasks and Measures**

Subjective Responses

1. Profile of Mood States – (POMS; McNair et al. 1971) The POMS is a validated measure consisting of 72 adjectives commonly used to describe momentary mood states. The POMS is highly sensitive to the effects of drugs in similar samples of healthy volunteers (Johanson & Uhlenhuth 1980), and will be used to assess mood effects of the drug during the study sessions.
2. The Addiction Research Center Inventory (ARCI; Martin et al. 1971): The 49-item ARCI is a true-false questionnaire with five empirically-derived scales that are sensitive to the effects of a variety of classes of abused drugs including amphetamine, marijuana and alcohol.
3. Drug Effects Questionnaire - (DEQ; Fischman & Foltin 1991) The DEQ is a validated measure consisting of questions on a visual analog scale about the subjective effects of drugs. Subjects are asked to rate the extent they feel a drug effect, whether they like or dislike the drug effect, and if given a choice would they want to take more of the drug. This is also be used to assess the pharamcodynamics of the drug effect during the study

Objective Demand for Drugs: To measure objective demand for the drugs, participants will complete a previously validated Multiple Choice procedure (Griffiths et al. 1993; Griffiths et al. 1996). In this procedure participants make choices between receiving the drug they just got again vs. receiving varying amounts of money. Monetary amounts are arranged in ascending order, and increment such that each value is 1.1x the previous value. The value at which the participant switches fro choosing drug to choosing money is referred to as the “crossover point”, and is the primary dependent variable. The range of monetary amounts will be $0.50 - $30, which has previously been shown to capture the crossover point for similar doses of d-amphetamine and THC. Ideally the Multiple Choice Procedure would be administered at a separate session during which participants are not drug affected, and would be paired with a real-life consequence, i.e. one of the hypothetical choices would be randomly selected and administered to the participant. However, we do not have the resources in this initial pilot study to add additional sessions, so as a compromise we will use a hypothetical version administered at the end of the study after peak drug effect. The Multiple Choice procedure has been administered successfully under similar conditions previously (Alessi et al. 2003a; Greenwald & Stitzer 2000; Tancer & Johanson 2007), and hypothetical choices on behavioral economic measures have been shown to correlate very closely with consequenced choices (Johnson & Bickel 2002), so we believe this will still yield a valid measure of demand. Estimated total yearly income will be collected during screening for use as a covariate in analyses of this measure.

Emotional Responses to External Stimuli

1. International Affective Picture System (IAPS) – (Lang et al. 1999) Participants will view standardized positive, negative and neutral pictures from the IAPS. The negative and positive images will be matched on degree of valence and arousal. An Evaluative Space Grid rating screen measuring subjective positive, negative and arousal reactions to the pictures will follow each picture. This procedure has previously been shown to capture effects of alcohol and amphetamine on emotional responses (Stritzke et al. 1995; Wardle & de Wit 2012), although as noted above, THC had no effect (Ballard et al. 2012).
2. Dynamic Emotion Identification Task (DEIT) – In a task created for use in our laboratory (Wardle et al. 2012), participants will view dynamically developing facial expressions composed of 2% morphs between a neutral face and a 100% expression of an emotion presented sequentially. The emotions will be happiness, sadness, anger, fear and disgust. Participants will be instructed to respond as soon as they believe they can correctly identify the emotion expressed. In this way sensitivity to external emotional stimuli (% of emotion expressed at time of identification) can be measured. Accuracy is usually near ceiling with this paradigm, and thus is not a primary dependent variable. This and similar face recognition tasks have been shown to be sensitive to effects of alcohol, amphetamine and THC (Attwood et al. 2009; Ballard et al. 2012; Wardle et al. 2012)

Impulsivity: Impulsivity is a complex and multi-faceted construct (Reynolds et al. 2006). We are not able to include a full battery of impulsivity tasks in this initial pilot study, but have selected a validated measure of impulsive action, the Cued Go/No Go Task as our initial starting point. In this task, at the beginning of each trial, a cue is presented in the form of a horizontal or vertical rectangle. The orientation of the rectangle indicates the probability that the following target will be a go target, to which participants should respond, or a no-go target, to which they should not respond. Horizontal cues signal a go trial 80% of the time, and a no-go 20% of the time, while vertical cues signal a no-go 80% of the time and a go cue 20% of the time. After a random interval ranging from 100ms-500ms, cues are followed by the appearance of either a green go target, to which the participants must press the “/” key on the keyboard, or a blue no-go target, to which the participant must make no response. To encourage quick and accurate responding, participants receive “correct” or “incorrect” feedback after each trial, along with their reaction time in ms. The primary measure is the proportion of no-go trials on which the participant fails to inhibit a response. We chose this task because it is sensitive to the inhibition-impairing effects of alcohol. In particular alcohol-induced impairments on this task are related both to chronic binge drinking, and choosing to drink more ad-lib during a laboratory challenge (Marczinski et al. 2007; Weafer & Fillmore 2008), suggesting this task captures key effects of drugs on impulsivity that can lead to increased drug consumption. However, as noted above, amphetamine actually decreases impulsivity on this same task (de Wit et al. 2002), providing a strong test for divergent effects.

Cardiovascular measures: Blood pressure and heart rate will be periodically monitored using portable blood pressure cuffs.

**Data Analysis:** We use three distinct placebo sessions to avoid regression to the mean artificially inflating correlations, which would occur if we used a single placebo session as our comparison point for all three drugs.

Manipulation Check: For each of our measures (subjective responses, objective demand, emotional responses, impulsivity and cardiovascular), we will first examine whether each drug had a significant overall effect on the measure compared to its unique placebo session, using three one-way ANOVAs with drug as the independent variable (alcohol vs. placebo, amphetamine vs. placebo and THC vs. placebo). We will summarize variables that are assessed multiple times across the study (subjective responses, cardiovascular variables), using peak change scores relative to baseline. At this stage we will also examine the relationship of potential covariates, including weight, age, drug use history and income with our measures, and select the variables with significant effects on our DVs as covariates for the relevant drug effect analyses.

Comparing Drug Effects: We will then construct “Drug Effect Scores” on each variable for each drug by subtracting the score on the unique placebo session for that drug from the score from the drug session. We will conduct 3 regressions on each measure examining the relationship between: 1. alcohol and amphetamine responses 2. alcohol and THC responses, and 3. THC and amphetamine responses. We will control for any selected covariates in these regressions. In the event that more than two drugs correlate on a given measure, we will use the Intraclass Correlation Coefficient (ICC) to estimate the degree of overlap between all three drugs (Shrout 1998; Shrout & Fleiss 1979). On an exploratory basis, we will also examine relationships across our several measures of drug effects (i.e. subjective, demand, emotional response, impulsivity and cardiovascular variables) within drug conditions, using regressions and controlling for relevant covariates. It is comparatively rare for multiple drug response variables from these different categories to all be included in a single study, and thus important information about their relationships may be gained from this exploratory analysis. We do not have the N in this preliminary pilot study to conduct a full factor analysis of our response variables, but we hope to gain information that could be used to guide a future factor analysis examining which of these response variables are driven by related vs. independent underlying mechanisms.

**Human Subjects Information**

**Recruiting methods**: We will place print ads in newspapers and on online job search sites such as craigslist.org, and flyer in the Chicago area. Healthy volunteers who respond to our ads are screened using our standard screening protocol for all studies in the Human Behavioral Psychopharmacology Laboratory, which is separately approved by the IRB under Protocol #13681B

**Obtaining consent:** Written informed consent for the screening session only is obtained at the screening according to procedures outlined in Protocol #13681B. Written informed consent for the study procedures is obtained at the orientation session, after a verbal explanation of study procedures, check of comprehension, and an opportunity for the participant to ask any questions they may have. Consent is verbally re-verified at the beginning of each study session.

**Risk to subjects:**

1. Diagnostic procedures and questionnaires: Some of the questions asked during the screening may be considered sensitive information, including drug use history and psychiatric history. We have rigorous procedures in place to ensure confidentiality of data, including locked cabinets for confidential files, subject coding, secure computer systems, and rigorous training of personnel. Please see screening protocol #13681B for full information on steps taken to protect information gathered as part of the screening.

2. Study drugs: There are some general precautions we take that apply to all drugs used in the study. We reduce the risk of side effects from the administered drugs by using low doses, and separating individual doses by at least five full days (study sessions will be separated by at least two full days, but because placebo and drug sessions are interspersed, drug sessions will be separated by five days). Volunteers are screened for both physical and psychiatric contraindications, as described above. The drugs are administered in a hospital setting with medical assistance readily available. Heart rate and blood pressure are monitored at frequent intervals during the study, our study physician is always on call, and the emergency room is minutes away. Subjects are told not to drive for 12 hours following sessions and, if necessary, are reimbursed for public transportation costs. Subjects are also provided with phone numbers to contact the study investigators and study physician in the event of an adverse reaction developing after the end of the session. Subjects will be told that small amounts of the drugs or their metabolites could be detectable in the body for several weeks and to advise the experimenter if they intend to undergo a drug screening within one month of participating in the study. Drug-specific risks and precautions are described below:

A. Alcohol. Potential effects of alcohol include dizziness, irritability, confusion, drowsiness, sleepiness, sleep disturbance, lightheadedness, nausea, vomiting, loss of coordination and clumsiness, gait disturbance, lack of concentration, tremor, restlessness, gastrointestinal upset, headache, pallor, flushing, sweating, dry mouth, slurred speech and fatigue. Rare side effects that could be more serious include: depression, itching, fever, palpitation (heart pounding), arrhythmia (irregular heart beats), nausea, increased or decreased blood pressure, blurred vision, rash, or possibly an allergic reaction. The dose selected is unlikely to cause these adverse effects in participants such as ours who are carefully screened for psychiatric and medical problems, and who report current (past month) use of a roughly equivalent dose. Furthermore, several previous studies have safely administered these doses using similar procedures (i.e., 0.8 g/kg administered within 15 minutes) to a wide range of alcohol users, including light drinkers (less than 5 drinks/week) as well as moderate and heavy drinkers (e.g. Doty & de Wit 1995; Evans & Levin 2004; King et al. 2011). Participants will not be cleared to leave the study until their BAL is below 0.04 on two consecutive breath tests, per NIAAA Guidelines. This is well within the legal limit for driving in Illinois (< 0.08) and is not expected to be impairing. Nevertheless, participants will be told not to drive for 12 hours following the study, and will be provided with compensation if they need to take public transit. Given our precautions and results from previous research, we believe that administration of alcohol in the context of this study poses a low risk to participants.

B. Amphetamine. Side effects of amphetamine include allergic reactions, irregular heartbeat or high blood pressure, headache, blurred vision, hallucinations, abnormal behavior, or confusion, restlessness or tremor, anxiety or nervousness, insomnia, dry mouth or an unpleasant taste in the mouth, diarrhea or constipation, impotence or changes in sex drive. Most participants do not experience any of these effects, and when they do occur they are short-lived. The most common side effect we have observed under laboratory conditions is transiently elevated blood pressure or heart rate. We have established specific cardiovascular guidelines in cooperation with our physician, and automatically contact the study physician in the event of these limits being exceeded during our regular monitoring of blood pressure and heart rate. We have given 20mg doses of d-amphetamine under these same conditions in many other studies in our laboratory (e.g. Brauer & de Wit 1995; Childs & de Wit 2009; de Wit et al. 1997; de Wit et al. 2002; de Wit et al. 1986; Hart et al. 2012b), with no adverse events. Given our precautions and results from previous research, we believe that administration of amphetamine in the context of this study poses a low risk to participants.

C. THC. The possible side effects of THC include: Constipation, drowsiness, coordination problems, memory loss, tiredness, depression, dizziness, faintness, rapid heart rate, raised blood pressure, restlessness, dry mouth, changes in sex drive, double or blurred vision, confusion shakiness, tremor, headache, nausea, muscle weakness, changes in appetite, difficulty sleeping, feelings of fear, changes in vision or hearing, addiction. However, the risk of these effects is very low at the doses used in this study. We apply the same precautions detailed above for alcohol and amphetamine in our THC studies, including careful cardiovascular monitoring and instructions to participants not to drive for 12 hours after the study. We have given 7.5mg doses, and even higher (up to 15mg) doses of THC under these same conditions in other studies in our laboratory with no adverse effects (e.g. Ballard et al. 2012; McDonald et al. 2003; Wachtel & de Wit 2000).

The risk of addiction created by administration of these drugs is low in the current study for the following reasons: 1) the drug is administered in a controlled setting (that is, a clinical laboratory), 2) subjects are only exposed to drugs a limited number of times, 3) there is no evidence to suggest that administration of a drug in a medical setting (for medication or research purposes) leads to abuse of that drug in a non-medical setting (College on Problems of Drug Dependence 1995; Schuster 1989). Furthermore, the participants we recruit are occasional users of recreational drugs, and there is evidence that most of these occasional users never go on to use drugs excessively (Sbriglio et al. 1988). Volunteers are excluded from our studies if there is any indication of stimulant dependence, either current or in the past. Several investigators who conduct studies involving administration of drugs have followed up their subjects to monitor drug use, and there has been no evidence of increased use after participation (Bigelow et al. 1995; Kaufman et al. 2000). Thus, we believe that it is very unlikely that subjects exposed to drugs in our medical/laboratory setting after careful screening will increase their use, go on to abuse those drugs in a recreational setting, or to increase their level of recreational drug use as a result of their participation. Despite the low likelihood of this risk, we will advise subjects about the risk for abuse in the consent form.

Because the subjects are normal healthy adults participating voluntarily, there are no alternative treatments. They will be fully debriefed following the study.

3. Tasks: Some of the tasks (emotional pictures, pictures of facial expressions) employ stimuli that are designed to elicit short-term positive and negative emotional reactions. Although the pictures used are designed to elicit emotional reactions, these reactions are typically brief, and similar methods to have previously been used in a wide range of studies without evidence of any long-term adverse reactions. Further, participants are screened for any psychiatric conditions that might make them vulnerable to experiencing adverse reactions to brief alterations in mood. Any participants who are unduly distressed will be counseled by a trained staff member.

**Benefits to subjects:** There is no direct benefit to the participants, although we hope that the information learned from this study will contribute to our knowledge of factors influencing drug use. Additionally, participating in research may be an educational experience for participants, and we attempt to facilitate this by providing a thorough debriefing including an explanation of study hypotheses and procedures at the conclusion of participation.

**Subject time commitment and compensation:** The screening portion of the study takes approximately 2 hours. The orientation typically takes approximately 1 hour. The study sessions are estimated to last 5.5 hrs each, and the debriefing is .5 hours, for a total of 36.5 hours spent in study sessions. Participants are compensated $30 for each of the 6 study sessions, with a bonus of $170 for completion of all study sessions, giving a total of $350.

**Data and Safety Monitoring:** The PI will monitor data collection and safety at weekly staff meetings. During these meetings, the PI will review and respond appropriately to (1) data collection and storage practices and (2) any adverse or unexpected effects from the study drugs. Both the study physician and PI will monitor the safety of study participants on an ongoing basis. The physician connected with this study will be on call during the experimental sessions and for 24 hours after sessions. Subjects will be given telephone numbers for the study physician and investigators in case they experience unpleasant effects after leaving the laboratory.

If an unanticipated problem were to occur, the staff member most closely involved with the subject at that time or the physician would notify the PI immediately. The PI would then submit written notification of the problem to the problem to the IRB using the “Unanticipated Problem” report within 10 working days. The PI would then determine, in collaboration with the IRB whether the problem requires further reporting to the federal funding agency or FDA. If a life-threatening adverse event were to occur, the PI would communicate the event to the IRB chair immediately, and halt further study sessions and participant enrollment.

**References**

Alessi SM, Greenwald M, Johanson C-E (2003a) The prediction of individual differences in response to D-amphetamine in healthy adults. Behavioural Pharmacology 14: 19-32

Alessi SM, Greenwald M, Johanson CE (2003b) The prediction of individual differences in response to D-amphetamine in healthy adults. Behavioural Pharmacology 14: 19-32

Attwood AS, Ohlson C, Benton C, Penton-Voak IS, Munafò MR (2009) Effects of acute alcohol consumption on processing of perceptual cues of emotional expression. Journal of Psychopharmacology 23: 23-30

Badiani A, Belin D, Epstein D, Calu D, Shaham Y (2011) Opiate versus psychostimulant addiction: The differences do matter. Nat Rev Neurosci 12: 685-700

Ballard ME, Bedi G, de Wit H (2012) Effects of delta-9-tetrahydrocannabinol on evaluation of emotional images. Journal of Psychopharmacology 26: 1289-1298

Bigelow GE, Brooner RK, Walsh SL, Preston KL, Liebson I (1995) Community outcomes following research exposure to cocaine or opiods. In: Harris LS (ed) Problems of Drug Dependence 1994: Proceedings of the 56th Annual Scientific Meeting (NIDA Research Monograph 153). US Government Printing Office, Washington, D.C., pp 354

Brauer LH, de Wit H (1995) Role of dopamine in d-amphetamine-induced euphoria in normal, healthy volunteers. Experimental and Clinical Psychopharmacology 3: 371-381

Childs E, de Wit H (2009) Amphetamine-Induced Place Preference in Humans. Biological Psychiatry 65: 900-904

College on Problems of Drug Dependence (1995) Human subject issues in drug abuse research. Drug and Alcohol Dependence 37: 167-175

Curran V, Brignell C, Fletcher S, Middleton P, Henry J (2002) Cognitive and subjective dose-response effects of acute oral Δ9-tetrahydrocannabinol (THC) in infrequent cannabis users. Psychopharmacology 164: 61-70

de Wit H, Clark M, Brauer LH (1997) Effects of d-Amphetamine in Grouped Versus Isolated Humans. Pharmacology Biochemistry and Behavior 57: 333-340

de Wit H, Doty P (1994) Preference for ethanol and diazepam in light and moderate social drinkers: a within-subjects study. Psychopharmacology 115: 529-538

de Wit H, Enggasser JL, Richards JB (2002) Acute administration of d-amphetamine decreases impulsivity in healthy volunteers. Neuropsychopharmacology 27: 813-825

de Wit H, Phillips TJ (2012) Do initial responses to drugs predict future use or abuse? Neuroscience &amp; Biobehavioral Reviews 36: 1565-1576

de Wit H, Uhlenhuth EH, Johanson CE (1986) Individual differences in the reinforcing and subjective effects of amphetamine and diazepam. Drug and Alcohol Dependence 16: 341-360

Doty P, de Wit H (1995) Effect of setting on the reinforcing and subjective effects of ethanol in social drinkers. Psychopharmacology 118: 19-27

Evans SM, Levin F (2004) Differential response to alcohol in light and moderate female social drinkers. Behavioural pharmacology 15: 167-181

Fischman MW, Foltin RW (1991) Utility of subjective-effects measurements in assessing abuse liability of drugs in humans. British Journal of Addiction 86: 1563-1570

Frezza M, di Padova C, Pozzato G, Terpin M, Baraona E, Lieber CS (1990) High Blood Alcohol Levels in Women. New England Journal of Medicine 322: 95-99

Greenwald MK, Stitzer ML (2000) Antinociceptive, subjective and behavioral effects of smoked marijuana in humans. Drug and Alcohol Dependence 59: 261-275

Griffiths R, Troisi JRI, Silvermian K, Miumford GK (1993) Multiple-choice procedure: an efficient approach for investigating drug reinforcement in humans. Behavioural Pharmacology 4: 3-14

Griffiths RR, Bigelow GE, Ator NA (2003) Principles of initial experimental drug abuse liability assessment in humans. Drug and Alcohol Dependence 70: S41-S54

Griffiths RR, Rush CR, Puhala KA (1996) Validation of the multiple-choice procedure for investigating drug reinforcement in humans. Experimental and Clinical Psychopharmacology 4: 97-106

Haberstick BC, Zeiger JS, Corley RP, Hopfer CJ, Stallings MC, Rhee SH, Hewitt JK (2011) Common and drug-specific genetic influences on subjective effects to alcohol, tobacco and marijuana use. Addiction 106: 215-224

Hart A, Wit H, Palmer A (2012a) Genetic Factors Modulating the Response to Stimulant Drugs in Humans. In: Cryan JF, Reif A (eds) Behavioral Neurogenetics (Current Topics in Behavioral Neurosciences). Springer Berlin Heidelberg, pp 537-577

Hart AB, Engelhardt BE, Wardle MC, Sokoloff G, Stephens M, de Wit H, Palmer AA (2012b) Genome-wide association study of *d*-amphetamine response in healthy volunteers identifies putative associations, including cadherin 13 (*CDH13*). PLoS ONE 7: e42646

Holdstock L, de Wit H (1999) Individual differences in subjective responses to ethanol and triazolam. Behavioural Pharmacology 10: 283-295

Holdstock L, de Wit H (2001) Individual differences in responses to ethanol and {d}-amphetamine: A within-subject study. Alcoholism: Clinical and Experimental Research 25: 540-548

Johanson CE, Uhlenhuth EH (1980) Drug preference and mood in humans: d-amphetamine. Psychopharmacology 71: 275-279

Johnson MW, Bickel WK (2002) WITHIN-SUBJECT COMPARISON OF REAL AND HYPOTHETICAL MONEY REWARDS IN DELAY DISCOUNTING. Journal of the Experimental Analysis of Behavior 77: 129-146

Kaufman MJ, Levin JM, Kukes TJ, Villafuerte RA, Hennen J, Lukas SE, Mendelson JH, Renshaw PF (2000) Illicit cocaine use patterns in intravenous-naive cocaine users following investigational intravenous cocaine administration. Drug and Alcohol Dependence 58: 35-42

Kedia S, Sell MA, Relyea G (2007) Mono-versus polydrug abuse patterns among publicly funded clients. Substance abuse treatment, prevention, and policy 2: 33

King AC, de Wit H, McNamara PJ, Cao D (2011) Rewarding, stimulant, and sedative alcohol responses and relationship to future binge drinking. Archives of general psychiatry 68: 389

Lang PJ, Bradley MM, Cuthbert BN (1999) International affective picture system (IAPS): Technical manual and affective ratings. NIMH Center for the study of emotion and attention, University of Florida, NIMH Center for the study of emotion and attention, University of Florida

Marczinski CA, Combs SW, Fillmore MT (2007) Increased sensitivity to the disinhibiting effects of alcohol in binge drinkers. Psychology of Addictive Behaviors 21: 346-354

Martin WR, Sloan JW, Sapira JD, Jasinski DR (1971) Physiologic, subjective and behavioral effects of amphetamine, methamphetamine, ephedrine, phenmetrazine, an methylphenidate in man. Clinical Pharmacology and Therapeutics 12: 245-258

McDonald J, Schleifer L, Richards JB, de Wit H (2003) Effects of THC on behavioral measures of impulsivity in humans. Neuropsychopharmacology

McKim WA, Hancock SD (2003) Drugs and behavior: An introduction to behavioral pharmacology. Prentice Hall, Prentice Hall

McNair D, Lorr M, Droppleman L (1971) Profile of Mood States. Educational and Industrial Testing Service, Educational and Industrial Testing Service

Nestler EJ (2005) Is there a common molecular pathway for addiction? Nature Neuroscience 8: 1445-1449

Perkins KA, Fonte C, Ashcom J, Broge M, Wilson A (2001) Subjective responses to nicotine in smokers may be associated with responses to caffeine and to alcohol. Experimental and clinical psychopharmacology 9: 91-100

Reynolds B, Ortengren A, Richards JB, de Wit H (2006) Dimensions of impulsive behavior: Personality and behavioral measures. Personality and Individual Differences 40: 305-315

Sbriglio R, Hartman N, Millman RB, Khuri ET (1988) Drug and alcohol abuse in children and adolescents Handbook of clinical assessment of children and adolescents, Vols. 1 & 2. New York, NY, US: New York University Press, pp 915-937

Schuster CR (1989) Testing and abuse liability of drugs in humans. In: Fischman MW, Mello NK (eds) Testing for abuse liabilty of drugs in humans (NIDA Research Monograph No. 92). National Institute of Drug Abuse, Rockville, MD, pp 1- 6

Sher KJ, Walitzer KS (1986) Individual differences in the stress-response-dampening effect of alcohol: A dose-response study. Journal of Abnormal Psychology 95: 159-167

Shrout PE (1998) Measurement reliability and agreement in psychiatry. Statistical methods in medical research 7: 301

Shrout PE, Fleiss JL (1979) Intraclass correlations: Uses in assessing rater reliability. Psychological Bulletin 86: 420-428

Stoops WW, Fillmore MT, Poonacha MS, Kingery JE, Rush CR (2003) Alcohol Choice and Amphetamine Effects in Light and Moderate Drinkers. Alcoholism: Clinical and Experimental Research 27: 804-811

Stritzke WG, Patrick CJ, Lang AR (1995) Alcohol and human emotion: A multidimensional analysis incorporating startle-probe methodology. Journal of Abnormal Psychology 104: 114-122

Sutker PB, Tabakoff B, Goist Jr KC, Randall CL (1983) Acute alcohol intoxication, mood states and alcohol metabolism in women and men. Pharmacology Biochemistry and Behavior 18, Supplement 1: 349-354

Tancer M, Johanson C-E (2007) The effects of fluoxetine on the subjective and physiological effects of 3,4-methylenedioxymethamphetamine (MDMA) in humans. Psychopharmacology 189: 565-573

Wachtel SR, de Wit H (2000) Naltrexone does not block the subjective effects of oral Delta(9)-tetrahydrocannabinol in humans. Drug and Alcohol Dependence 59: 251-60

Wardle M, de Wit H (2012) Effects of amphetamine on reactivity to emotional stimuli. Psychopharmacology 220: 143-153

Wardle M, Garner M, Munafò M, de Wit H (2012) Amphetamine as a social drug: effects of *d*-amphetamine on social processing and behavior. Psychopharmacology: 1-12

Watson PE, Watson ID, Batt RD (1981) Prediction of blood alcohol concentrations in human subjects; updating the Widmark equation. Journal of Studies on Alcohol and Drugs 42: 547

Weafer J, Fillmore M (2008) Individual differences in acute alcohol impairment of inhibitory control predict ad libitum alcohol consumption. Psychopharmacology 201: 315-324

White TL, Justice AJH, de Wit H (2002) Differential subjective effects of *d*-amphetamine by gender, hormone levels and menstrual cycle phase. Pharmacology, Biochemistry and Behavior 73: 729-741

White TL, Lott DC, de Wit H (2006) Personality and the subjective effects of acute amphetamine in healthy volunteers. Neuropsychopharmacology 31: 1064-1074

Zeiger JS, Haberstick BC, Corley RP, Ehringer MA, Crowley TJ, Hewitt JK, Hopfer CJ, Stallings MC, Young SE, Rhee SH (2012) Subjective effects for alcohol, tobacco, and marijuana association with cross-drug outcomes. Drug and Alcohol Dependence 123, Supplement 1: S52-S58
