# Supplementary material for: A Preliminary Investigation of Individual Differences in Subjective Responses to D-Amphetamine, Alcohol, and Delta-9-Tetrahydrocannabinol Using a Within-Subjects Randomized Trial
Source: PLoS One. 2015 Oct 29;10(10):e0140501. doi: 10.1371/journal.pone.0140501 (PMC4626040; doi:10.1371/journal.pone.0140501)
Supplement: S1 Table — (DOCX) [file pone.0140501.s003.docx]

| Item 1  (Scale: Drug) | Mean | Std. Deviation | Item 2  (Scale: Drug) | Mean | Std. Deviation | *t* | *p* |
| --- | --- | --- | --- | --- | --- | --- | --- |
| Feel: AMP | 21.8333 | 26.04622 | Feel: ALC | 59.0000 | 23.94922 | -5.465 | <.001 |
| Feel: AMP | 21.8333 | 26.04622 | Feel: THC | 19.6250 | 33.44536 | .250 | .815 |
| Feel: ALC | 59.0000 | 23.94922 | Feel: THC | 19.6250 | 33.44536 | 4.841 | <.001 |
| Like: AMP | 37.2500 | 34.72782 | Like: ALC | 30.7083 | 37.18167 | .521 | .559 |
| Like: AMP | 37.2500 | 34.72782 | Like: THC | 3.4583 | 36.33417 | 3.010 | .004 |
| Like: ALC | 30.7083 | 37.18167 | Like: THC | 3.4583 | 36.33417 | 2.371 | .025 |
| Dislike: AMP | -2.0000 | 34.32580 | Dislike: ALC | 27.5833 | 38.68369 | -3.087 | .005 |
| Dislike: AMP | -2.0000 | 34.32580 | Dislike: THC | 9.8333 | 35.09821 | -1.014 | .306 |
| Dislike: ALC | 27.5833 | 38.68369 | Dislike: THC | 9.8333 | 35.09821 | 1.385 | .172 |
| High: AMP | 17.9583 | 27.15251 | High: ALC | 38.8750 | 29.92936 | -2.977 | .009 |
| High: AMP | 17.9583 | 27.15251 | High: THC | 18.7917 | 35.95405 | -.116 | .936 |
| High: ALC | 38.8750 | 29.92936 | High: THC | 18.7917 | 35.95405 | 1.998 | .052 |
| More: AMP | 31.2083 | 38.35870 | More: ALC | 14.7500 | 35.08406 | 1.638 | .109 |
| More: AMP | 31.2083 | 38.35870 | More: THC | -1.5000 | 30.56284 | 2.840 | .007 |
| More: ALC | 14.7500 | 35.08406 | More: THC | -1.5000 | 30.56284 | 1.379 | .170 |

S1 Table. Post-hoc t-tests comparing peak drug effects relative to placebo for each pair of drugs (AMP vs. ALC, AMP vs. THC, ALC vs. THC) on the DEQ "Feel," Like," "Dislike," and "Want More" scales.
