# Supplementary material for: A Preliminary Investigation of Individual Differences in Subjective Responses to D-Amphetamine, Alcohol, and Delta-9-Tetrahydrocannabinol Using a Within-Subjects Randomized Trial
Source: PLoS One. 2015 Oct 29;10(10):e0140501. doi: 10.1371/journal.pone.0140501 (PMC4626040; doi:10.1371/journal.pone.0140501)
Supplement: S3 Table — (DOCX) [file pone.0140501.s005.docx]

| AMP Feel | | | |  |  | ALC High |  |  | | |
| --- | --- | --- | --- | --- | --- | --- | --- | --- | --- | --- |
| Variable | β | SE | t | *p* |  | Variable | β | SE | t | *p* |
| Social Potency | .621 | .676 | .919 | .369 |  | Social Potency | -.586 | .795 | -.737 | .470 |
| Negative Emotionality | .226 | .751 | .301 | .766 |  | Negative Emotionality | .433 | .884 | .490 | .629 |
| Constraint | -.648 | .947 | -.685 | .501 |  | Constraint | .308 | 1.11 | .276 | .785 |
| *R^2^ =* .089 |  |  |  |  |  | *R^2^ =* .045 |  |  |  |  |
| *F*(3,20) =.652 | |  |  |  |  | *F*(3,20) =.314 |  |  |  |  |
| *p* =.591 |  |  |  |  |  | *p* =.815 |  |  |  |  |
|  |  |  |  |  |  |  |  |  |  |  |
| AMP Like |  |  |  |  |  | ALC More |  |  |  |  |
| Variable | β | SE | t | *p* |  | Variable | β | SE | t | *p* |
| Social Potency | 1.10 | .835 | 1.32 | .203 |  | Social Potency | .896 | .841 | 1.07 | .299 |
| Negative Emotionality | 1.37 | .928 | 1.48 | .154 |  | Negative Emotionality | -.617 | .934 | -.660 | .517 |
| Constraint | --.452 | 1.17 | -.387 | .703 |  | Constraint | -1.94 | 1.18 | -1.65 | .115 |
| *R^2^ =* .467 |  |  |  |  |  | *R^2^ =* .223 |  |  |  |  |
| *F*(3,20) =1.861 | |  |  |  |  | *F*(3,20) =1.913 |  |  |  |  |
| *p* =.169 |  |  |  |  |  | *p* =.160 |  |  |  |  |
|  |  |  |  |  |  |  |  |  |  |  |
| AMP Dislike |  |  |  |  |  | THC Feel |  |  |  |  |
| Variable | Β | SE | t | *p* |  | Variable | β | SE | t | *p* |
| Social Potency | -.260 | .872 | -.298 | .769 |  | Social Potency | -.474 | .890 | -.532 | .600 |
| Negative Emotionality | -1.16 | .969 | -1.20 | .244 |  | Negative Emotionality | .443 | .989 | .448 | .659 |
| Constraint | 1.35 | 1.22 | 1.11 | .282 |  | Constraint | .597 | 1.25 | .479 | .637 |
| *R^2^=* .356 |  |  |  |  |  | *R^2^=* .207 |  |  |  |  |
| *F*(3,20) =.966 | |  |  |  |  | *F*(3,20) =.297 |  |  |  |  |
| *p* = .428 |  |  |  |  |  | *p* = .827 |  |  |  |  |
|  |  |  |  |  |  |  |  |  |  |  |
| AMP High |  |  |  |  |  | THC Like |  |  |  |  |
| Variable | β | SE | t | *p* |  | Variable | β | SE | t | *p* |
| Social Potency | .578 | .734 | .703 | .490 |  | Social Potency | -1.58 | .899 | -1.76 | .094 |
| Negative Emotionality | -.617 | .815 | -.772 | .449 |  | Negative Emotionality | .499 | .998 | .500 | .623 |
| Constraint | -.061 | 1.028 | .161 | .873 |  | Constraint | .641 | 1.258 | .510 | .616 |
| *R^2^=* .232 |  |  |  |  |  | *R^2^=* .173 |  |  |  |  |
| *F*(3,20) = .379 | |  |  |  |  | *F*(3,20) = 1.40 |  |  |  |  |
| *p* = .769 |  |  |  |  |  | *p* = .273 |  |  |  |  |
|  |  |  |  |  |  |  |  |  |  |  |
| AMP More |  |  |  |  |  | THC Dislike |  |  |  |  |
| Variable | β | SE | t | *p* |  | Variable | β | SE | t | *p* |
| Social Potency | .890 | .972 | .916 | .371 |  | Social Potency | -.427 | .890 | -.480 | .636 |
| Negative Emotionality | .892 | 1.08 | .826 | .418 |  | Negative Emotionality | 1.66 | .989 | 1.682 | .108 |
| Constraint | -1.17 | 1.36 | -.856 | .402 |  | Constraint | -.987 | 1.25 | -.792 | .438 |
| *R^2^=* .132 |  |  |  |  |  | *R^2^=* .361 |  |  |  |  |
| *F*(3,20) = 1.019 | |  |  |  |  | *F*(3,20) = 1.00 |  |  |  |  |
| *p* = .409 |  |  |  |  |  | *p* = .413 |  |  |  |  |
|  |  |  |  |  |  |  |  |  |  |  |
| ALC Feel |  |  |  |  |  | THC High |  |  |  |  |
| Variable | β | SE | t | *p* |  | Variable | β | SE | t | *p* |
| Social Potency | -.707 | .618 | -1.14 | .266 |  | Social Potency | .175 | .965 | .182 | .858 |
| Negative Emotionality | .469 | .686 | .683 | .502 |  | Negative Emotionality | .181 | 1.07 | .169 | .868 |
| Constraint | -1.04 | .866 | -1.20 | .245 |  | Constraint | .865 | 1.35 | .640 | .530 |
| *R^2^=* .100 |  |  |  |  |  | *R^2^=* .025 |  |  |  |  |
| *F*(3,20) =.737 | |  |  |  |  | *F*(3,20) = .174 |  |  |  |  |
| *p* = .542 |  |  |  |  |  | *p* = .913 |  |  |  |  |
|  |  |  |  |  |  |  |  |  |  |  |
| ALC Like |  |  |  |  |  | THC More |  |  |  |  |
| Variable | β | SE | t | *p* |  | Variable | β | SE | t | *p* |
| Social Potency | -.050 | .836 | -.060 | .952 |  | Social Potency | -1.34 | .774 | -1.73 | .100 |
| Negative Emotionality | -.512 | .929 | -.552 | .587 |  | Negative Emotionality | .159 | .859 | .185 | .855 |
| Constraint | -3.215 | 1.17 | -2.75 | .012 |  | Constraint | -.339 | 1.08 | -.313 | .758 |
| *R^2^=* .316 |  |  |  |  |  | *R^2^=* .133 |  |  |  |  |
| *F*(3,20) = 3.08 | |  |  |  |  | *F*(3,20) = 1.026 |  |  |  |  |
| *p* = .051 |  |  |  |  |  | *p* = .402 |  |  |  |  |
| ALC Dislike |  |  |  |  |  |  |  |  |  |  |
| Variable | β | SE | t | *p* |  |  |  |  |  |  |
| Social Potency | -.652 | 1.01 | -.649 | .524 |  |  |  |  |  |  |
| Negative Emotionality | -.135 | 1.12 | -.121 | .905 |  |  |  |  |  |  |
| Constraint | 1.391 | 1.41 | .988 | .335 |  |  |  |  |  |  |
| *R^2^=* .087 |  |  |  |  |  |  |  |  |  |  |
| *F*(3,20) = .632 |  |  |  |  |  |  |  |  |  |  |
| *p* = .603 |  |  |  |  |  |  |  |  |  |  |

S3 Table. Linear regression model of three MPQ scales (Negative Emotionality, Constraint, and Social Potency) and their contribution to the effects of AMP, ALC, and THC on the DEQ.
